# Circular RNA *Cdr1as* inhibits proliferation and delays injury-induced regeneration of the intestinal epithelium

**DOI:** 10.1172/jci.insight.169716

**Published:** 2024-01-16

**Authors:** Hee Kyoung Chung, Lan Xiao, Naomi Han, Jason Chen, Vivian Yao, Cassandra M. Cairns, Benjamin Raufman, Jaladanki N. Rao, Douglas J. Turner, Rosemary Kozar, Myriam Gorospe, Jian-Ying Wang

**Affiliations:** 1 Cell Biology Group, Department of Surgery, University of Maryland School of Medicine, Baltimore, Maryland, USA.; 2 Baltimore Veterans Affairs Medical Center, Baltimore, Maryland, USA.; 3 Shock Trauma Center, University of Maryland School of Medicine, Baltimore, Maryland, USA.; 4 Laboratory of Genetics and Genomics, National Institute on Aging-IRP, NIH, Baltimore, Maryland, USA.; 5 Department of Pathology, University of Maryland School of Medicine, Baltimore, Maryland, USA.

**Keywords:** Gastroenterology, Surgery

## Abstract

Circular RNAs (circRNAs) are highly expressed in the mammalian intestinal epithelium, but their functions remain largely unknown. Here, we identified the circRNA *Cdr1as* as a repressor of intestinal epithelial regeneration and defense. *Cdr1as* levels increased in mouse intestinal mucosa after colitis and septic stress, as well as in human intestinal mucosa from patients with inflammatory bowel disease and sepsis. Ablation of the *Cdr1as* locus from the mouse genome enhanced renewal of the intestinal mucosa, promoted injury-induced epithelial regeneration, and protected the mucosa against colitis. We found approximately 40 microRNAs, including miR-195, differentially expressed between intestinal mucosa of *Cdr1as*-knockout (*Cdr1as^–/–^*) versus littermate mice. Increasing the levels of *Cdr1as* inhibited intestinal epithelial repair after wounding in cultured cells and repressed growth of intestinal organoids cultured ex vivo, but this inhibition was abolished by miR-195 silencing. The reduction in miR-195 levels in the *Cdr1as^–/–^* intestinal epithelium was the result of reduced stability and processing of the precursor miR-195. These findings indicate that *Cdr1as* reduces proliferation and repair of the intestinal epithelium at least in part via interaction with miR-195 and highlight a role for induced *Cdr1as* in the pathogenesis of unhealed wounds and disrupted renewal of the intestinal mucosa.

## Abstract 2

<p>CircRNA <em>Cdrlas</em> reduced injury-induced regeneration of the intestinal epithelium by deregulating miR-195.</p>

## Graphical abstract

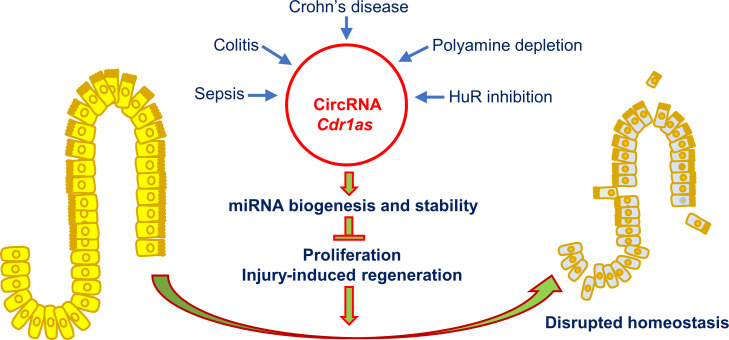

## Introduction

The mammalian intestinal mucosa elicits a spectrum of responses after acute injury and repairs itself rapidly to restore epithelial integrity (1–3). The repair of the intestinal mucosa after injury is a complex process consisting of epithelial restitution characterized by stages of cell spreading and migration into the wounded area, replacement of lost cells by cell proliferation, and epithelial remodeling via differentiation (3–5). Successful repair of wounds and ulcers requires that intestinal epithelial cells (IECs) change gene expression patterns to modulate cell survival, migration, proliferation, and differentiation (6–8). Gut mucosal injury occurs commonly in patients with inflammatory bowel disease (IBD) and critical surgical disorders such as sepsis, trauma, and massive thermal injuries (9–11). Disrupted intestinal epithelial integrity by severe and diffusive injury/erosions results in the translocation of luminal toxic substances and bacteria to the bloodstream, sometimes leading to multiple organ dysfunction syndrome and death (11, 12). Effective therapies to preserve the integrity of the intestinal epithelium and enhance recovery of damaged mucosa in patients with critical illnesses are limited to date, at least in part because the mechanisms underlying gut mucosal injury and repair are poorly understood.

The intestinal epithelium expresses a large number of circular RNAs (circRNAs) that are mainly produced from precursor RNAs undergoing back-splicing (13, 14). Unlike linear RNAs, such as mRNAs and most long noncoding RNAs (lncRNAs), circRNAs are covalently closed at the 5′ and 3′ ends, rendering them particularly stable (15). Evidence is accumulating that circRNAs regulate a wide variety of biological processes and are intimately involved in human pathologies through diverse mechanisms, including interactions with microRNAs (miRNAs) and RNA-binding proteins (RBPs), regulation of transcription and splicing, and translation into small peptides (15, 16). We recently reported that approximately 300 circRNAs, including *Cdr1as* and *circHIPK3*, were differentially expressed in the damaged mucosa of the small intestine in mice exposed to septic stress induced by cecal ligation and puncture (CLP) (14). Increasing *circHIPK3* levels enhanced intestinal epithelial repair after wounding and promoted constant mucosal renewal primarily by acting as a sponge for miRNA-29b (miR-29b). However, the function of most circRNAs enriched in the intestinal epithelium is unknown, and elucidating their roles might be exploited to benefit patients with critical illnesses.

The circRNA *Cdr1as* is a circularized antisense transcript of the *Cdr1* gene (which encodes cerebellar degeneration–related protein 1) and is highly conserved across mammals (17, 18). *Cdr1as* is not detectable as a linear transcript in human and mouse cells because it is efficiently circularized (16, 18). Human *Cdr1as* harbors more than 70 binding sites for miR-7 and acts as a sponge for miR-7 by reducing the number of freely available miR-7 molecules (16, 18). *Cdr1as* also has a full complementary binding site for miR-671, but the interaction between *Cdr1as* and miR-671 was found to slice *Cdr1as* (19). Although the basal levels of *Cdr1as* are relatively low in many tissues and organs, its expression levels change remarkably in human pathologies such as cancers and inflammation (14, 20). Genetic ablation of the *Cdr1as* locus from the mouse genome decreased miR-7 levels and derepressed production of miR-7–regulated proteins in brain, thus affecting brain function (21). However, no studies so far have investigated the role of *Cdr1as* in regulating intestinal epithelium homeostasis, particularly its implication in gut mucosal injury and repair. Here, we provide evidence that *Cdr1as* levels in the intestinal mucosa increased markedly in mice with colitis or exposed to septic stress and in the intestinal mucosa of human patients with injury/erosions and inflammation from IBD and sepsis. We further discovered that *Cdr1as*-knockout (*Cdr1as^–/–^*) mice displayed enhanced mucosal defense and injury-induced epithelial regeneration in the intestine, and this protective function by *Cdr1as* deletion was at least partially mediated by reducing miR-195 in these tissues.

## Results

### Changes in Cdr1as levels in mouse and human intestinal epithelium responding to stress.

**Figure 1:**
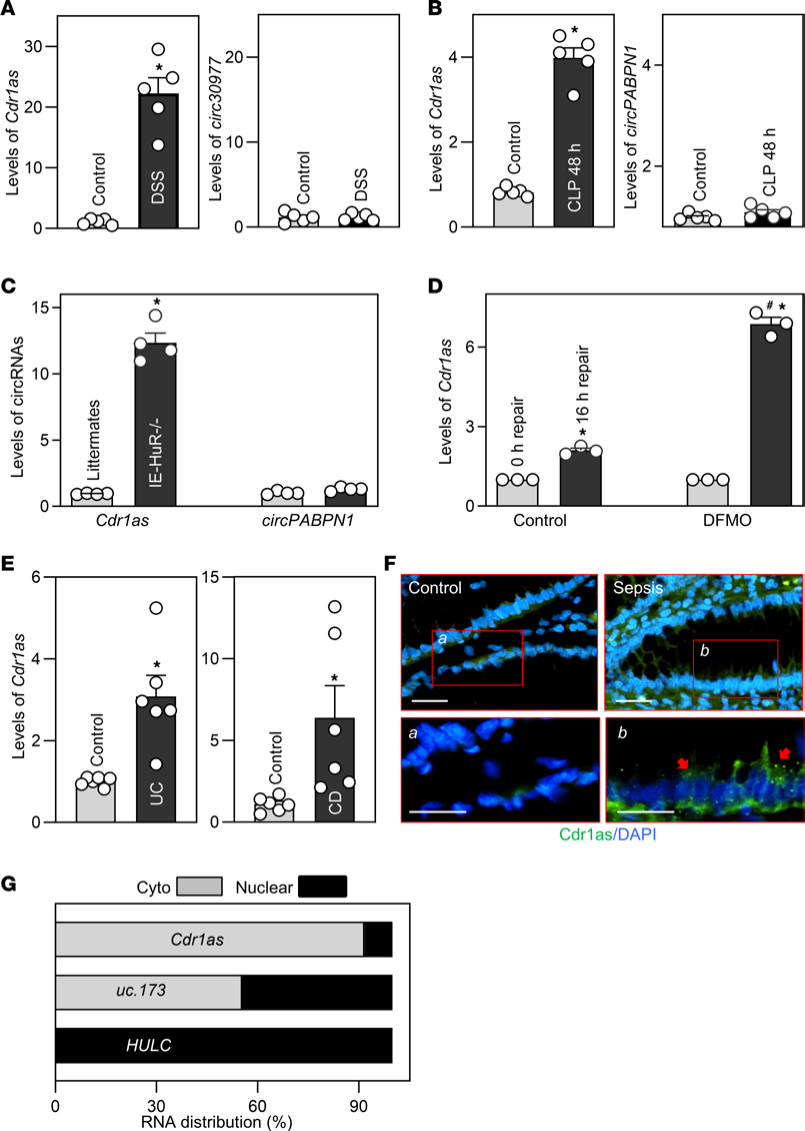
Mucosal *Cdr1as* expression in the intestine associated with various pathologies. (**A**) Levels of mucosal *Cdr1as* and *circ30977* in the colons of mice treated with water (control) or 3% DSS for 7 days as measured using Q-PCR. Values are the mean ± SEM (*n* = 5). *P < 0.05 compared with control mice. (**B**) Levels of mucosal *Cdr1as* and *circPABPN1* in the small intestine of mice exposed to CLP for 48 hours (*n* = 5). *P < 0.05 compared with control. (**C**) Levels of mucosal *Cdr1as* and *circPABPN1* in the small intestine from control littermate and IE-HuR^–/–^ mice (*n* = 4). *P < 0.05 compared with littermates. (**D**) Levels of *Cdr1as* in cultured Caco-2 cells 16 hours after wounding in the presence or absence of cellular polyamines. Cells were exposed to DFMO (5 mmol/L) for 6 days, and the levels of *Cdr1as* were examined 16 hours after wounding (*n* = 3). *P < 0.05 and ^#^*P* < 0.05 compared with 0-hour repair and 16-hour repair of the control group, respectively. (**E**) Mucosal *Cdr1as* levels in the colon from patients with ulcerative colitis (UC) and the ileum from patients with Crohn disease (CD) (*n* = 6). *P < 0.05 compared with controls. (**F**) In situ hybridization of *Cdr1as* with fluorescent LNA-RNA detection probe in the small intestinal mucosa in patients with sepsis, as shown in green. Scale bars: 25 μm; arrows indicate *Cdr1as* staining. Experiments were conducted in 4 control individuals and 4 septic patients and showed similar results. (**G**) Levels of cytoplasmic (cyto) and nuclear *Cdr1as*, *uc.173*, and *HULC* in Caco-2 cells. Statistical significance was analyzed using unpaired, 2-tailed Student’s *t* test, except for results in **F** and **G**. All experiments in **F** and **G** were repeated 3 times with similar results.

Two murine models, dextran sulphate sodium–induced (DSS-induced) mucosal inflammatory injury in colon (22) and CLP-induced mucosal injury in small intestine (23), were used to examine the involvement of *Cdr1as* in intestinal mucosal injury in this study. As reported in our previous work (24) and that of others (25), after we administered 3% DSS in their drinking water for 7 days, mice exhibited colonic mucosal granulocyte infiltration, epithelial damage, and bloody diarrhea, mimicking the injury/erosions observed in human ulcerative colitis. As shown in Figure 1A, the mucosal *Cdr1as* level increased dramatically in the colons of DSS-treated mice, achieving greater than 20-fold higher levels than those observed in control animals; this increase in *Cdr1as* abundance was specific, since DSS treatment did not alter the colon mucosal levels of other circRNAs such as *circ30977*. Consistent with previous studies (14, 23), exposure of mice to CLP for 48 hours induced mucosal lesions in the small intestine, as indicated microscopically by severely sloughed cells, denuded villi with dilated capillaries, and hemorrhage, and macroscopically by bleeding and edematous and swollen mucosa with areas of red streaks. Exposure to CLP for 48 hours also resulted in an acute gut barrier dysfunction, as evidenced by an increased mucosal permeability to fluorescein isothiocyanate–dextran (FITC-dextran), as reported previously (26, 27). In CLP mice, induced mucosal injury/erosions in the small intestinal mucosa were also associated with a substantial increase in tissue levels of *Cdr1as* without affecting the abundances of mucosal *circPABPN1* (Figure 1B). The latter finding again supports the specificity of the increase in *Cdr1as* in response to small intestinal mucosal injury.

The RNA-binding protein (RBP) HuR is a biological regulator of the intestinal epithelium homeostasis, whereas tissue-specific deletion of HuR (IE-HuR^–/–^) in mice delayed healing of intestinal mucosa after acute injury (22) and led to gut barrier dysfunction (28), which represents other pathological conditions. As shown in Figure 1C, IE-HuR^–/–^ mice also displayed a remarkable increase in the levels of mucosal *Cdr1as* in the small intestine, compared with those observed in control littermates. On the other hand, there were no significant differences in the levels of intestinal mucosal *circPABPN1* when comparing IE-HuR^–/–^ mice with littermate animals.

Polyamines are essential for maintaining the integrity of the intestinal epithelium, and decreasing the levels of cellular polyamines inhibits the growth of the intestinal mucosa and slows the repair of the intestinal epithelium after injury (29). In an in vitro intestinal epithelial injury model using cultured human colorectal adenocarcinoma–derived Caco-2 cells, the delayed epithelial repair induced by polyamine depletion was also accompanied by an increase in the cellular abundance of *Cdr1as* (Figure 1D). Consistent with our prior studies (30), treatment of Caco-2 cells for 6 days with D,L-α-difluoromethylornithine (DFMO), a specific inhibitor of polyamine biosynthesis (30), almost completely depleted the cellular polyamines putrescine and spermidine. Polyamine depletion by DFMO inhibited intestinal epithelial repair after wounding, as indicated by a significant decrease in cells over the wounded area at 16 hours, similar to our previous findings (29). Interestingly, decreasing the levels of cellular polyamines with DFMO increased *Cdr1as* content during epithelial repair after wounding. The levels of *Cdr1as* increased marginally 16 hours after wounding in control cells, but this induction was markedly enhanced in polyamine-deficient cells. These findings support a role for *Cdr1as* in polyamine-regulated epithelial repair after wounding.

To determine the implication of altered *Cdr1as* clinically, we measured the levels of *Cdr1as* in human intestinal mucosal tissues from patients with critical intestinal illnesses. Colonic and small intestinal mucosal tissues from patients with ulcerative colitis (UC), Crohn disease (CD), and sepsis were collected for measurements of *Cdr1as* levels; tissue samples from patients without gut mucosal injury/erosions and inflammation served as controls. The intestinal mucosal tissues obtained from all patients with UC, CD, and sepsis (Figure 1, E and F) displayed remarkable increases in the levels of *Cdr1as* when compared with those observed in controls. RNA fluorescence in situ hybridization (RNA-FISH) detection in the mucosa of the small intestine from septic patients showed that the elevated *Cdr1as* was not only localized in the epithelium but also accumulated in the submucosal lamina propria, and that in epithelial cells, *Cdr1as* was predominantly cytoplasmic (Figure 1F). The increased levels of mucosal *Cdr1as* in patients with UC, CD, and sepsis were associated with severe mucosal inflammation, injury/erosions, and delayed healing, as we reported previously (14, 28).

In Caco-2 cells, *Cdr1as* was mainly localized in the cytoplasm (Figure 1G); as controls, we also measured the levels of the nuclear lncRNA *HULC*, and lncRNA *uc.173*, localized in both the cytoplasm and the nucleus (31). Together, the data collected from mice, cultured IECs, and human tissues indicate that expression levels of *Cdr1as* in the intestinal epithelium increase in various gut mucosal pathologies, supporting its important role in the response to different stresses.

### Deletion of the Cdr1as locus promotes renewal and defense of the intestinal epithelium.

**Figure 2:**
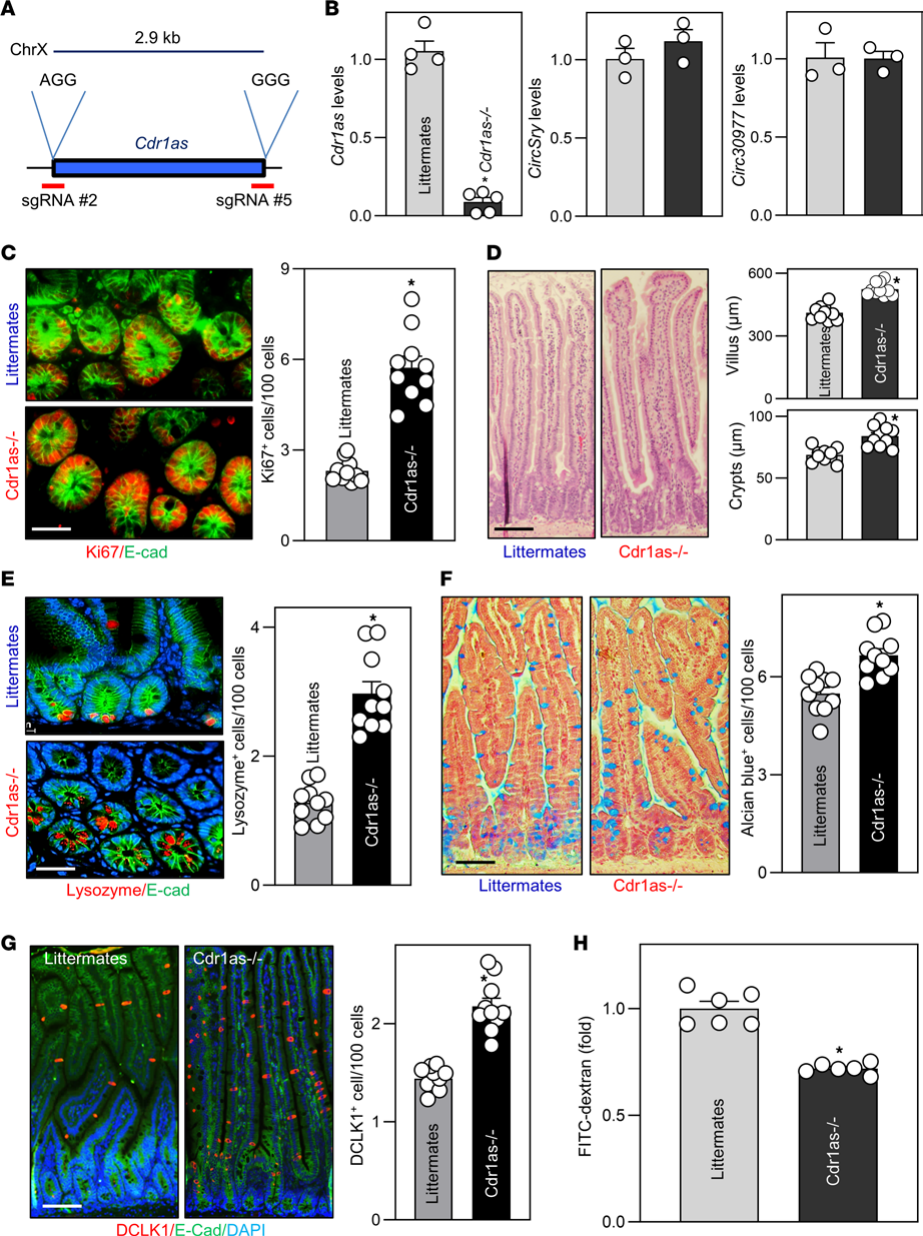
Targeted deletion of the *Cdr1as* locus by CRISPR/Cas9 promotes renewal of the intestinal epithelium and enhances gut barrier function in mice. (**A**) The sequences given denote protospacer-adjacent motifs. kb, kilobases. (**B**) Q-PCR assay confirmed successful genetic ablation of *Cdr1as*, as shown by a specific decrease in levels of *Cdr1as* in the small intestinal mucosa of *Cdr1as^–/–^* mice. Values are the mean ± SEM (*n* = 3 or 5). *P < 0.05 compared with littermates. (**C**) Left: Proliferating cells in small intestinal crypts in control littermate and *Cdr1as^–/–^* mice, as measured by Ki67 immunostaining. Red, Ki67; green, E-cadherin (E-cad). Right: Quantitative data of Ki67-positive cells (*n* = 10). *P < 0.05 compared with littermates. (**D**) Photomicrographs of H&E (left) and changes in the length of villi and crypts (right) of the mucosa described in **C** (*n* = 10). *P < 0.05 compared with littermates. (**E**–**G**) Changes in Paneth cells (lysozyme-positive cells), goblet cells (Alcian blue staining), and tuft cells (DCLK1-positive cells) in the mucosa described in **C**. *P < 0.05 compared with littermates (*n* = 10). (**H**) Changes in gut permeability in littermate and *Cdr1as^–/–^* mice. FITC-dextran was given orally, and blood samples were collected 4 hours thereafter for measurement. *P < 0.05 compared with littermates (*n* = 6). In **B**–**H**, statistical significance was analyzed using unpaired, 2-tailed Student’s *t* tests. Scale bars: 25 μm.

As *Cdr1as* is so efficiently circularized in mammals that it cannot be detected as a linear transcript (18, 19), the most straightforward strategy to explore the in vivo function of *Cdr1as* in the intestinal epithelium was to create a loss-of-function mouse model for this circRNA by ablating the *Cdr1as* locus from the mouse genome (Figure 2A and Supplemental Figure 1; supplemental material available online with this article; https://doi.org/10.1172/jci.insight.169716DS1) using CRISPR/Cas9, as described previously (19). Quantitative real-time PCR (Q-PCR) analysis revealed that *Cdr1as* levels in the intestinal mucosa (Figure 2B) and brain (Supplemental Figure 2A) were almost undetectable in our newly generated *Cdr1as^–/–^* mice, without an effect on the levels of other circRNAs such as *circSry* and *circ30977* (Figure 2B). *Cdr1as^–/–^* mice looked normal and were fertile and exhibited no significant abnormalities in body weight (Supplemental Figure 1E), gastrointestinal gross morphology (Supplemental Figure 1F), or general appearance compared with littermate controls.

Importantly, *Cdr1as^–/–^* mice displayed a substantial increase in the proliferating crypt cell population, marked by Ki67 immunostaining in the small intestine (Figure 2C), and longer villi and crypts (Figure 2D). Analysis of intestinal stem cell (ISC) activity further revealed that the small intestinal mucosa exhibited increased numbers of OLFM4- and LGR5-positive cells in *Cdr1as^–/–^* mice relative to littermates (Supplemental Figure 2B), suggesting that ISCs were activated in the *Cdr1as*-deficient intestinal epithelium. Decreasing the levels of *Cdr1as* in the intestinal epithelium also promoted the function of Paneth cells (Figure 2E), goblet cells (Figure 2E), and tuft cells (Figure 2G). Staining of whole mounts of the small intestine revealed that in control littermate mice, lysozyme-positive cells (Paneth cells) were located at the base of the crypt areas, but Alcian blue–positive cells (goblet cells) and double cortin–like kinase 1–positive (DCLK1-positive) cells (tuft cells) were distributed at both villous and crypt regions. The numbers of Paneth cells, goblet cells, and tuft cells in the small intestinal mucosa increased significantly in *Cdr1as^–/–^* mice relative to control littermates by approximately 110%, approximately 15%, and approximately 33%, respectively (Figure 2, E–G). However, *Cdr1as* deletion failed to alter enterocyte differentiation in the small intestinal mucosa, as examined by villin immunostaining analysis (data not shown). We also examined the levels of transcription factors responsible for differentiation of the secretory and absorptive epithelial cell lineages, and found that *Cdrlas* deletion did not alter the abundances of POU2F3, HES1, ATOH1, or SOX9 proteins in the small intestinal mucosa (Supplemental Figure 2C). These results suggest that *Cdr1as* may regulate the function of Paneth, goblet, and tuft cells by altering the overall renewal of the epithelium, rather than by specifically stimulating these transcriptional factors. Moreover, targeted deletion of the *Cdr1as* locus improved gut barrier function, since *Cdr1as^–/–^* mice exhibited a lower basal level of gut permeability than that observed in control littermates (Figure 2H), as measured by FITC-dextran assays (32).

**Figure 3:**
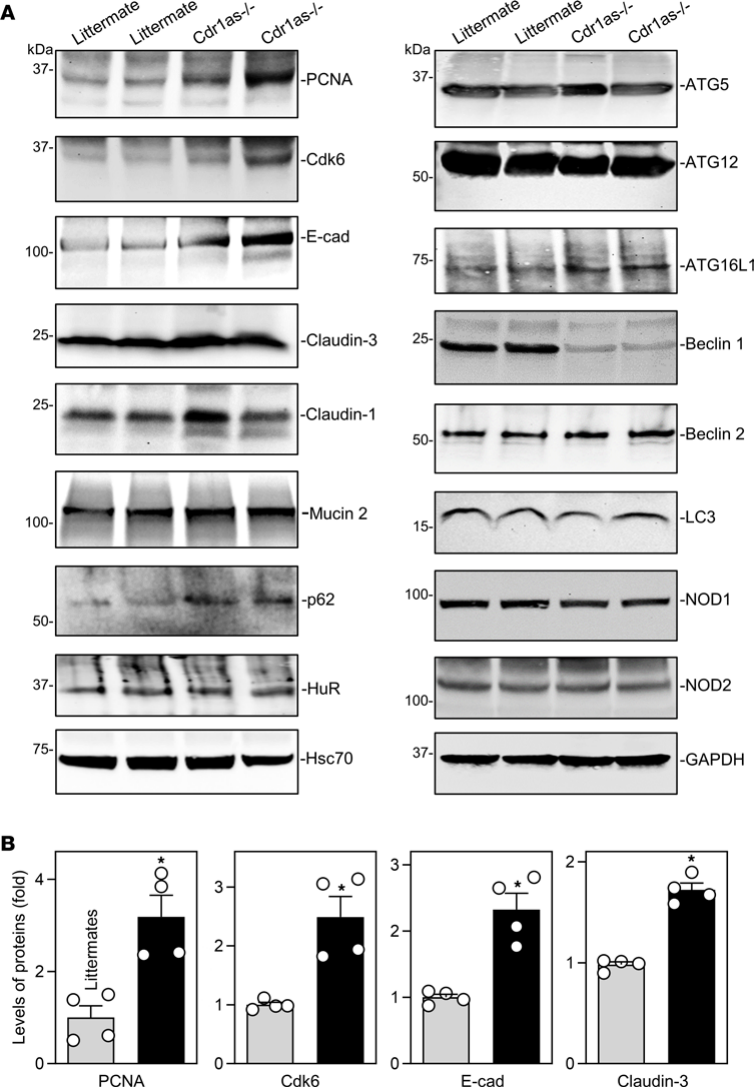
*Cdr1as* deletion increases the levels of proliferation-associated proteins and intercellular junctions in the intestinal epithelium. (**A**) Immunoblots of PCNA, Cdk6, E-cadherin (E-cad), occludin, mucin 2, RBPs (p62 and HuR), and autophagy-relevant proteins such as ATGs and their regulatory proteins in the small intestinal mucosa of littermate and *Cdr1as^–/–^* mice. Total proteins were isolated from the mucosal tissues and prepared for Western blot analysis. Equal loading was monitored by GAPDH. Three separate experiments were performed and showed similar results. (**B**) Quantitative analysis derived from densitometric scans of immunoblots of PCNA, Cdk6, E-cad, and Claudin-3 as described in **A**. Values are the mean ± SEM (*n* = 4). *P < 0.05 compared with control littermates. In **B**, statistical significance was analyzed using unpaired, 2-tailed Student’s *t* tests.

Consistent with its effect on the epithelial renewal and gut barrier function, *Cdr1as^–/–^* mice displayed increased levels of proliferation-associated proteins PCNA and Cdk6, adherens junction protein E-cadherin, and tight junction proteins claudin-3 and claudin-1 in the small intestinal mucosa, as measured by immunoblotting analysis (Figure 3, A and B). *Cdr1as* deletion failed to affect the levels of tight junction proteins occludin, ZO-1, and JAM-1 (Supplemental Figure 3A) and goblet cell–secreted protein mucin 2, but it increased the abundance of the RBP p62 without affecting HuR levels (Figure 3, A and B, and Supplemental Figure 3B). Since autophagy is crucial for host defense of the intestinal epithelium against invasive pathogens and for gut barrier function (26, 33), we also examined changes in the levels of autophagy-related proteins in the *Cdr1as*-deficient epithelium. Loss of *Cdr1as* did not alter the levels of ATG5, ATG12, ATG16L1, beclin 2, LC3, Hsc70, NOD1, and NOD2 in the small intestinal mucosa, but it did substantially reduce beclin 1 levels in *Cdr1as^–/–^* mice (Figure 3A and Supplemental Figure 3B). Another line of *Cdr1as^–/–^* mice generated from an independent founder was also examined and showed similar increases in intestinal mucosal growth and defense.

**Figure 4:**
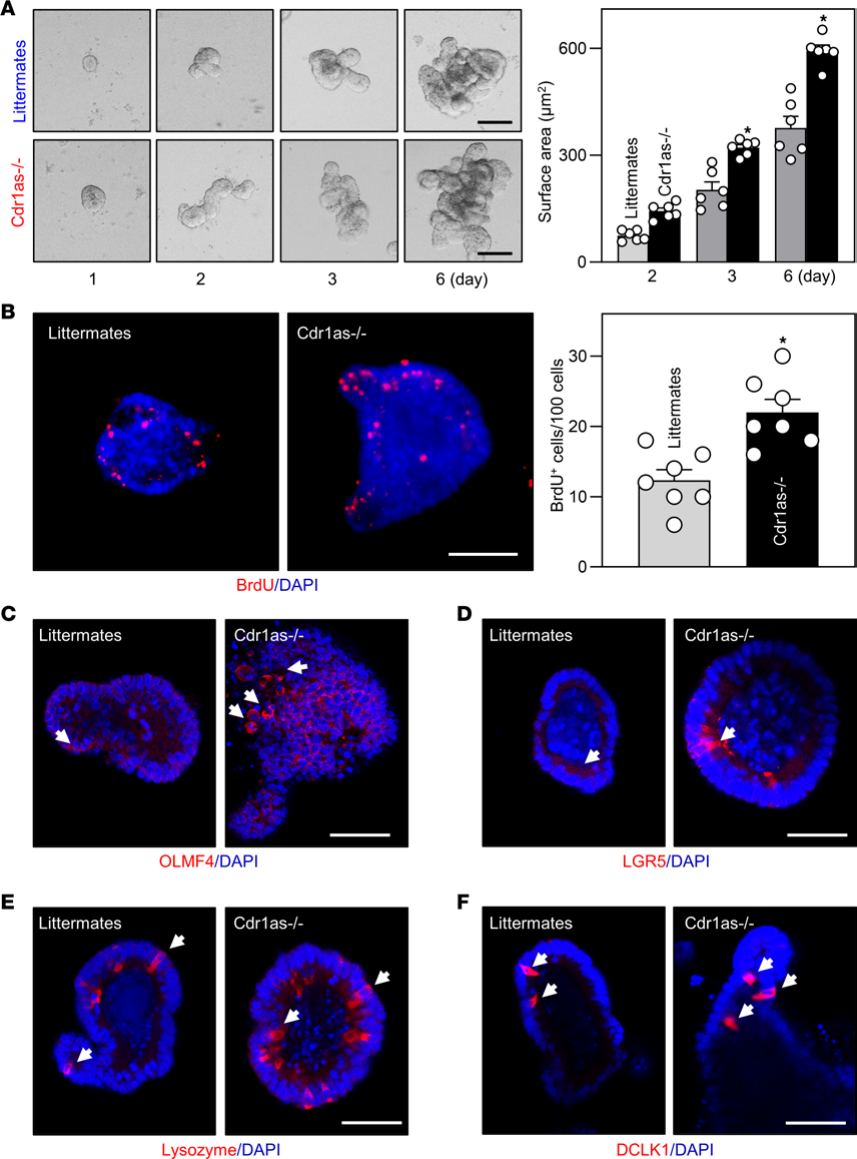
*Cdr1as* deletion increases the growth of intestinal organoids. (**A**) Growth of intestinal organoids derived from the proximal small intestine of *Cdr1as^–/–^* and control littermate mice. Images were taken at different times in culture. Values are the mean ± SEM (*n* = 6). *P < 0.05 compared with control littermates. (**B**) Proliferating cells in intestinal organoids on day 3 in culture, as measured by BrdU labeling (red). *P < 0.05 compared with control littermates (*n* = 6). (**C** and **D**) Activity of ISCs in intestinal organoids described in **B**, as measured by immunostaining using markers OLFM4 and LGR5. (**E** and **F**) Changes in Paneth cells and tuft cells in intestinal organoids on day 4 in culture, as marked by lysozyme and DCLK1, respectively. Scale bars: 50 μm. In **A** and **B**, statistical significance was analyzed using unpaired, 2-tailed Student’s *t* test.

To determine the involvement of secreted factors originating from stromal or immune cells of the gut in regulation of the intestinal mucosal growth after *Cdr1as* deletion, we examined the growth of intestinal organoids generated from *Cdr1as^–/–^* mice. Using methods described previously (8, 24), intestinal organoids were derived from proliferating crypts of the mucosa of the mouse small intestine. By 3 days after initial culture, the structures of intestinal organoids from both littermates and *Cdr1as^–/–^* mice consisted of multiple buds and cells (Figure 4A). However, intestinal organoids derived from *Cdr1as^–/–^* mice grew much faster than those from littermate mice, as evidenced by a substantial increase in the intestinal organoid surface area (Figure 4A) and BrdU incorporation (Figure 4B) in *Cdr1as*-deficient organoids compared with organoids generated from control littermates. The activity of ISCs in the *Cdr1as*-deicient intestinal organoids also increased markedly, as examined by immunohistochemical detection of ISC markers OLMF4 and LGR5 (Figure 4, C and D). In agreement with this observation, intestinal organoids derived from *Cdr1as^–/–^* mice exhibited increased numbers of Paneth and tuft cells when compared with those observed in the organoids from littermate mice (Figure 4, E and F, and Supplemental Figure 3C). However, there were no significant differences in goblet cells between *Cdr1as*-deficient organoids and control organoids, partially because of low basal numbers of mucin 2–positive cells when examined at 4 days into primary culture (Supplemental Figure 3D). These results indicate that the accelerated renewal of intestinal epithelial cells in *Cdr1as^–/–^* mice results primarily from a cell-autonomous regulatory effect of this circRNA and not from the effect of secreted factors in the gut. Together, the results from these in vivo and ex vivo experiments support the notion that *Cdr1as* is a negative regulator of the intestinal epithelium homeostasis.

### Loss of Cdr1as enhances epithelial repair after injury and protects the mucosa against colitis.

**Figure 5:**
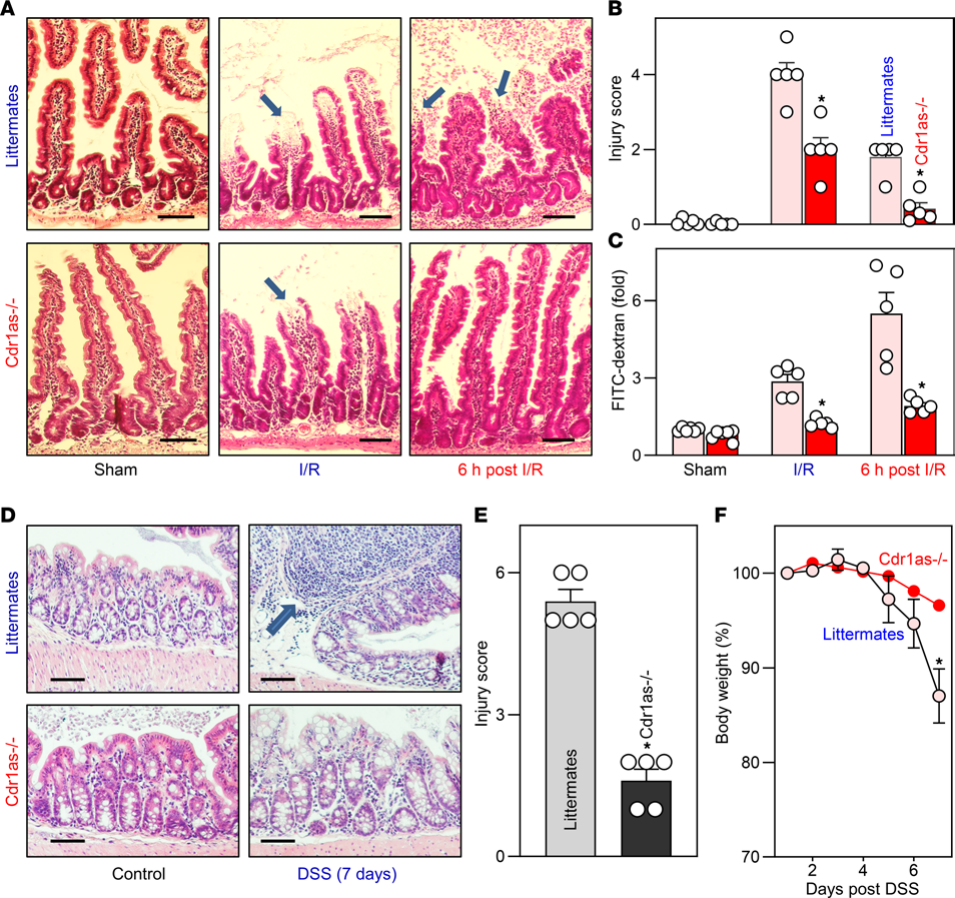
Targeted deletion of *Cdr1as* enhances injury-induced epithelial regeneration and protects the mucosa against colitis in mice. (**A**) Micrographs of the small intestinal mucosa from sham, I/R (mesenteric ischemia for 30 minutes, followed by reperfusion for 2 hours), and 6 hours after I/R in littermates and *Cdr1as^–/–^* mice. Arrow indicates injury and erosion. (**B**) Quantitation of the mucosal injury data in mice described in **A**. Values are the mean ± SEM (*n* = 5). *P < 0.05 compared with littermates. (**C**) Gut permeability in mice described in **A**. FITC-dextran was given orally, and blood samples were collected 4 hours later. *P < 0.05 compared with littermates. (**D**) Micrographs of the colonic mucosa in littermate and *Cdr1as^–/–^* mice treated with 3% DSS in drinking water for 7 days (DSS). Arrow indicates mucosal inflammatory injury. (**E**) Quantitation of the mucosal injury data in mice described in **D**. Values are the mean ± SEM (*n* = 5). *P < 0.05 compared with control. (**F**) Changes in body weights of mice described in **D**. *P < 0.05 compared with control (*n* = 5). In **B**, **C**, and **E**, statistical significance was analyzed using unpaired, 2-tailed Student’s *t* tests. In **F**, statistical comparison between time-course curves was by 2-way ANOVA with Bonferroni’s post hoc test. Scale bars: 25 μm.

To examine the role of *Cdr1as* in mucosal injury and repair in the small intestine, a mesenteric ischemia/reperfusion–induced (I/R-induced) mucosal injury model was used in this study. Both littermates and *Cdr1as^–/–^* mice subjected to mesenteric I/R exhibited signs of remarkable mucosal injury and erosions in the small intestine, as indicated by severe sloughed cells, denuded villi with dilated capillaries, and by frank hemorrhage microscopically (Figure 5A). Deletion of the *Cdr1as* locus decreased the degrees of I/R-induced mucosal injury, since the injury scores in *Cdr1as^–/–^* mice were lower than those observed in littermates when measured immediately after mesenteric I/R (Figure 5B). Moreover, *Cdr1as*-deficient intestinal epithelium displayed a substantial increase in mucosal repair after acute injury. In *Cdr1as^–/–^* mice, the mucosa repaired quickly, and the epithelial integrity was almost completely restored 6 hours after I/R. In contrast, the mucosal surface still showed some sloughed cells and debris at the same time after I/R in littermate mice. Consistent with these findings, loss of *Cdr1as* also enhanced the recovery of gut barrier function after I/R-induced stress, as evidenced by a rapid decrease in the gut permeability to FITC-dextran in *Cdr1as^–/–^* mice compared with littermate mice when examined 6 hours after I/R (Figure 5C). These results indicate that decreasing the levels of *Cdr1as* not only protects the mucosa against I/R-induced injury, but also enhances repair of damaged mucosa in the small intestine.

To determine the role of *Cdr1as* in colonic mucosal injury, DSS-induced colitis was used as a model. Similar to the observations in wild-type mice described in Figure 1A, control littermate mice also effectively developed mucosal injury/erosions and inflammation in the colon after administration of 3% DSS in drinking water for 7 days, as characterized by diffusive epithelial damage and granulocyte infiltration (Figure 5D, upper panel). However, *Cdr1as* deletion protected the colonic mucosa against DSS-induced injury, since there were only minor mucosal injury/erosions observed in *Cdr1as^–/–^* mice on day 7 after treatment with 3% DSS in drinking water (Figure 5D, lower panel). The sum of histological scores in the colonic mucosa decreased by approxmately 80% in *Cdr1as^–/–^* mice compared with littermates (Figure 5E). Deletion of *Cdr1as* also protected the colonic mucosa against DSS-induced apoptosis, as revealed by TUNEL staining (Supplemental Figure 4). Exposure to DSS for 7 days induced remarkable apoptosis in littermate mice, but *Cdr1as^–/–^* mice exhibited a substantial decrease in the rate of apoptotic cell death compared with littermate controls. In addition, both littermate and *Cdr1as^–/–^* mice began to lose weight after day 5 of DSS treatment, but this body weight loss was markedly slower in *Cdr1as^–/–^* mice relative to littermate animals (Figure 5F). These results indicate that *Cdr1as* inhibition protects the colonic mucosa against DSS-induced colitis.

### Cdr1as affects injury-induced epithelial regeneration via miR-195.

**Figure 6:**
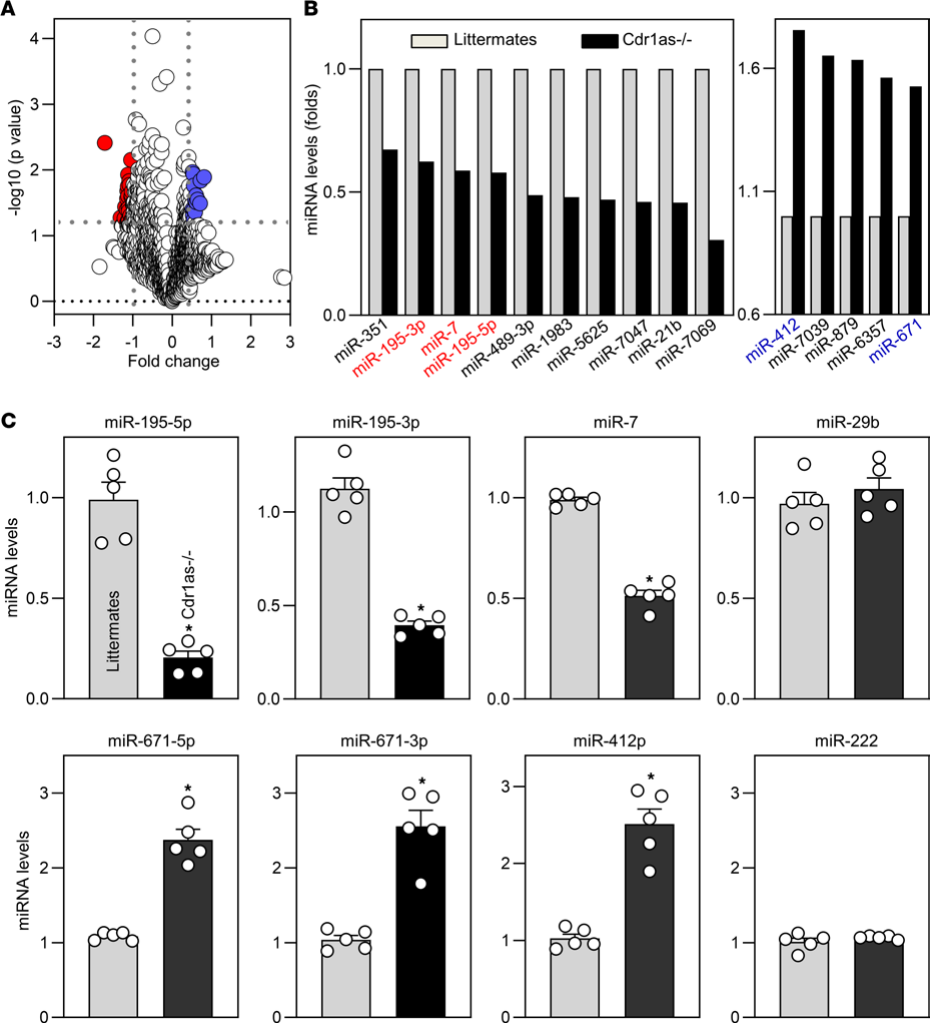
Changes in miRNA expression profile in the intestinal mucosa after *Cdr1as* deletion. (**A**) Scatter plot depictions of miRNAs differentially expressed in the small intestinal mucosa in littermate and *Cdr1as^–/–^* mice, as measured by miRNA microarray. (**B**) Differential expression analysis of miRNAs in results described in **A**. Values are the mean from 3 animals. (**C**) Levels of mucosal miR-195 and other miRNAs in the small intestine as examined by Q-PCR analysis. Values are the mean ± SEM (*n* = 5). *P < 0.05 compared with littermates. In **C**, statistical significance was analyzed using unpaired, 2-tailed Student’s *t* test.

To examine whether the loss of *Cdr1as* affects intestinal epithelium homeostasis by modulating miRNA function, microarray-based interrogation of global miRNA expression profiles in the small intestinal mucosa was performed. A comparison of the miRNA expression profiles in *Cdr1as^–/–^* mice relative to control littermates showed that 46 miRNAs were differentially expressed in the mucosa (Figure 6A; primary data sets deposited in the NCBI Gene Expression Omnibus: GEO GSE250489). In *Cdr1as^–/–^* mice, 28 miRNAs decreased (including miR-351, miR-195-3p, miR-7, miR-195-5p, miR-489-3p, miR-1983, miR-5625, miR-7047, miR-21b, and miR-7069) and 18 miRNAs increased (including miR-412, miR-7039, miR-879, miR-6357, and miR-671), when compared with control littermate mice (Figure 6B). Reverse transcription (RT) followed by Q-PCR analysis was used to confirm these changes and showed that loss of *Cdr1as* significantly lowered the levels of miR-195 and miR-7 in the small intestinal mucosa, but it increased the abundances of miR-671 and miR-412 without affecting the levels of miR-29b and miR-222 (Figure 6C).

**Figure 7:**
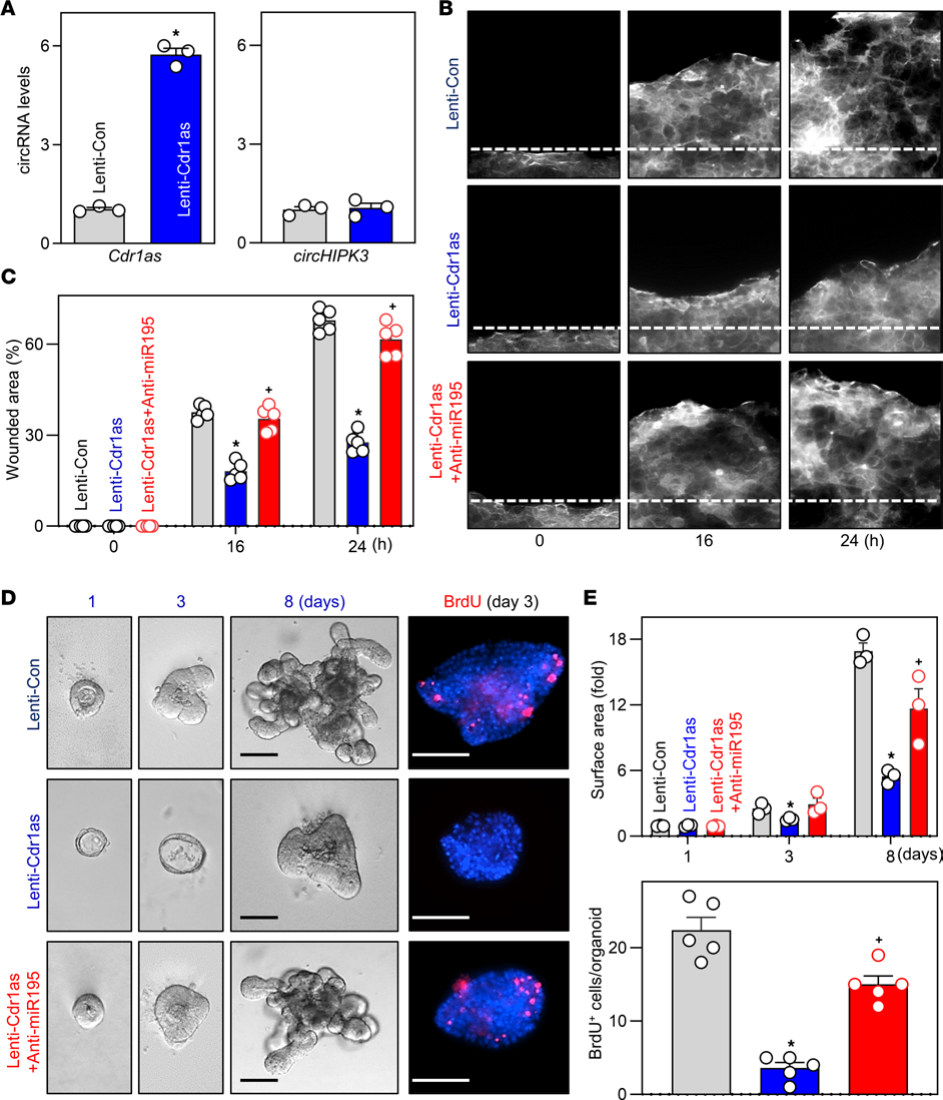
*Cdr1as* inhibits intestinal epithelial repair after wounding and decreases growth of intestinal organoids via miR-195. (**A**) Levels of *Cdr1as* and *circHIPK3* in Caco-2 cells 72 hours after infection with the lentiviral *Cdr1as* expression vector (Lenti-*Cdr1as*) or control lentiviral vector (Lenti-Con), as measured by Q-PCR. Values are the mean ± SEM (*n* = 3). *P < 0.05 compared with Lenti-Con. (**B**) Images of epithelial repair after wounding in cells described in **A**. The monolayer was wounded, and plates were photographed immediately, 16, and 24 hours thereafter. (**C**) Summarized data for cells described in **B**. Values are the mean ± SEM (*n* = 3). *P < 0.05 and ^+^*P* < 0.05 compared with Len-Con or Lenti-*Cdr1as* alone, respectively. (**D**) Growth of enteroids after *Cdr1as* overexpression ex vivo. Left: Bright-field microscopy analysis of enteroids sizes at different times after culture. Right: Confocal analysis of BrdU (red) and DAPI (blue) on day 3 after culture. Scale bars: 100 μm. (**E**) Quantification of surface area (*n* = 3) and BrdU-positive cells in enteroids described in **D**. *P < 0.05 and ^+^*P* < 0.05 compared with Len-Con or Lenti-*Cdr1as* alone, respectively. In **A**, statistical significance was analyzed using unpaired, 2-tailed Student’s *t* test, while comparisons of means between more than 2 groups were performed by 1-way ANOVA with Tukey’s multiple-comparison test for **C** and **E**.

To determine whether altered miRNAs plays a role in *Cdr1as*-regulated intestinal mucosal repair after wounding, we focused on miR-195 because it is highly expressed in the intestinal epithelium and influences mucosal growth (31, 34, 35) and epithelial repair after acute injury (36). An in vitro intestinal epithelial injury model using Caco-2 cells was used, as described previously (14, 36). The levels of *Cdr1as* were increased by infecting cells with a recombined lentiviral vector expressing *Cdr1as* (Lenti-*Cdr1as*) under the control of the suCMV promoter. By 72 hours after infection, the levels of cellular *Cdr1as* increased robustly and specifically (Figure 7A), while the abundance of another circRNA (*circHIPK3*) was not affected (Figure 7A). Inducing *Cdr1as* levels by Lenti-*Cdr1as* infection resulted in a remarkable increase in the levels of precursor miR-195 (pre-miR-195) and mature miR-195, but not miR-29b (Supplemental Figure 4B). As shown in Figure 7B, epithelial repair occurred quickly after wounding in cells infected with control lentiviral vector (Lenti-Con), as indicated by a significant decrease in the denuded area at 16 and 24 hours thereafter. Ectopic overexpression of *Cdr1as* by infection with Lenti-*Cdr1as* inhibited intestinal epithelial repair after wounding, and the numbers of cells over the wounded area were significantly lower in cells overexpressing *Cdr1as* than after infection with Lenti-Con (Figure 7, B and C). Importantly, neutralization of miR-195 by transfection with a miR-195 antagomir restored epithelial repair in *Cdr1as*-overexpressing cells, as evidenced by the fact that the numbers of cells over the wounded area in cells cotransfected with the Lenti-*Cdr1as* and anti–miR-195 were similar to those seen in the control group when examined at 16 and 24 hours after wounding (Figure 7, B and C). In addition, transfection with a miR-195 antagomir alone also modestly, but significantly, enhanced epithelial repair after wounding in cells infected with Lenti-Con (Supplemental Figure 4C).

To further study the effect of miR-195 on epithelial repair after wounding, we tested whether antagonizing miR-195 reversed the influence of *Cdr1as* on intestinal organoid growth ex vivo. As shown in Figure 7D, *Cdr1as* overexpression by infecting intestinal organoids with Lenti-*Cdr1as* markedly inhibited growth of intestinal organoids, as shown by reduced BrdU incorporation (Figure 7E) and robustly decreased intestinal organoid surface area (Figure 7E). Importantly, antagonizing miR-195 abolished the growth inhibition induced by *Cdr1as* overexpression in the Lenti-*Cdr1as* group, restoring the numbers of BrdU-positive cells and organoid surface area (Figure 7E) compared with those observed in organoids infected with Lenti-*Cdr1as* alone. These results indicate that *Cdr1as* reduces intestinal epithelial repair after wounding and inhibits mucosal renewal to a large extent by enhancing miR-195 function.

### Cdr1as regulates miRNA biogenesis and stabilizes pre-miR-195.

**Figure 8:**
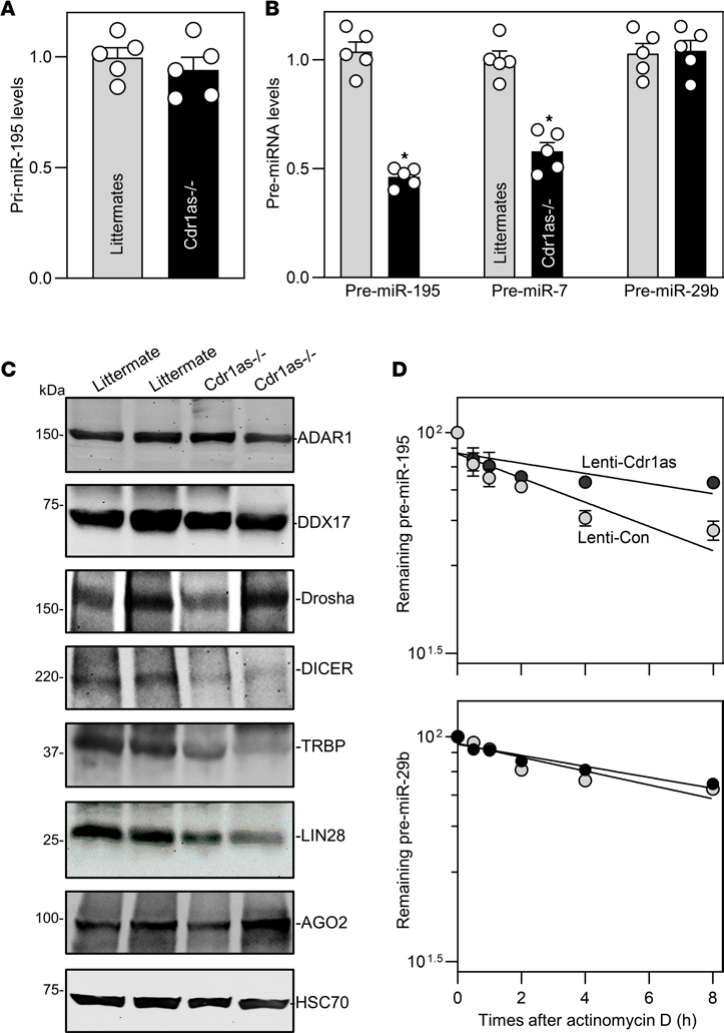
*Cdr1as* regulates miRNA biogenesis and stabilizes pre-miR-195 transcripts. (**A**) Levels of mucosal primary (pri-) miR-195 in the small intestine of littermate and *Cdr1as^–/–^* mice. Values are the mean ± SEM (*n* = 5). *P < 0.05 compared with littermates. (**B**) Levels of mucosal precursor (pre-) miR-195, pre-miR-7, and pre-miR-29b in the small intestine. *P <0.05 compared with littermates (*n* = 5). (**C**) Immunoblots of Drosha and Dicer complex proteins in the small intestinal mucosa of littermate and *Cdr1as^–/–^* mice. Equal loading was monitored by HSC70. (**D**) Stability of pre-miR-195 and pre-miR-29b after *Cdr1as* overexpression. Caco-2 cells were infected with Lenti-*Cdr1as* or Lenti-Con, and actinomycin D was added to the medium 72 hours after the infection. Levels of pre-miR-195 and pre-miR-29b were examined at different times thereafter. Three separate experiments were performed and showed similar results. In **A** and **B**, statistical significance was analyzed using unpaired, 2-tailed Student’s *t* tests. In **D**, statistical comparison between time-course curves was by 2-way ANOVA with Bonferroni’s post hoc test.

To define the mechanism by which loss of *Cdr1as* reduces miR-195 expression in the intestinal epithelium, the following 3 experiments were carried out in vivo as well as in vitro. First, we determined whether *Cdr1as* modulates miR-195 expression at the transcriptional level by examining changes in the levels of primary (pri-) and pre-miR-195 transcripts in the small intestinal mucosa of *Cdr1as^–/–^* and littermate mice. As shown in Figure 8A, loss of the *Cdr1as* locus in mice did not alter levels of tissue pri-miR-195, but it significantly decreased pre-miR-195 abundance in the intestinal mucosa (Figure 8B). The levels of mucosal pre-miR-195 in *Cdr1as^–/–^* mice were decreased by approximately 50% compared with control littermates. Like its effect on brain tissue (21), *Cdr1as* knockout also decreased the levels of pre-miR-7 in the intestinal mucosa, which served as a positive control in this study. In contrast, deletion of the *Cdr1as* locus did not alter the levels of pre-miR-29b in the intestinal mucosa. These results indicate that *Cdr1as* affects miR-195 expression through mechanisms other than transcription of pri-miR-195.

Second, we determined the involvement of *Cdr1as* in the regulation of miRNA processing in the intestinal epithelium. The levels of Drosha, Dicer, and their binding partners and regulatory proteins including ADAR1, DDX17, TRBP, LIN28, and Ago2 were examined in the small intestinal mucosa of *Cdr1as^–/–^* and control littermate mice. Drosha complex proteins ADAR1, DDX17, and Drosha were expressed at similarly high levels in the intestinal mucosa of control littermates and *Cdr1as^–/–^* mice (Figure 8C), suggesting that *Cdr1as* does not affect Drosha-mediated miRNA processing in the nucleus. Interestingly, targeted deletion of *Cdr1as* in mice reduced the Dicer complex, markedly lowering the levels of Dicer and TRBP proteins in the intestinal mucosa of *Cdr1as^–/–^* mice relative to control littermates (Figure 8C). In addition, *Cdr1as^–/–^* mice showed a selective decrease in LIN28 levels, but not in Ago2 levels, in the intestinal mucosa (Figure 8C). Because Dicer function is crucial for miRNA processing in the cytoplasm, a decrease in the levels of Dicer and TRBP proteins in the *Cdr1as*-deficient intestinal epithelium may contribute to the deregulation of miR-195 biogenesis.

Finally, we examined changes in the turnover of pre-miR-195 after *Cdr1as* overexpression. Ectopically overexpressed *Cdr1as* increased the levels of miR-195 (Supplemental Figure 4B) partially by altering pre-miR-195 stability, since the half-life of pre-miR-195 increased in cells infected with Lenti-*Cdr1as* relative to cells infected with Lenti-Con (Figure 8D), as measured by inhibiting de novo transcription using actinomycin D. This regulatory effect of *Cdr1as* on pre-miR-195 stability appeared to be specific, as the half-life of pre-miR-29b was not affected in cells overexpressing *Cdr1as* (Figure 8D). Together, these results strongly suggest that *Cdr1as* upregulates miR-195 expression at least partially by stabilizing pre-miR-195 and enhancing the processing from pre-miR-195 to mature miR-195 in the intestinal epithelium.

## Discussion

Hundreds of circRNAs are highly abundant in the mammalian intestinal epithelium and expressed in a tissue-specific manner (14, 33), but the functions of most circRNAs in gut mucosal biology and diseases remain largely unknown. In this study, we identified *Cdr1as* as a biological regulator of intestinal epithelium homeostasis by modulating mucosal growth and injury-induced regeneration and found that *Cdr1as* expression levels changed markedly in intestinal mucosal pathologies. Deletion of the *Cdr1as* locus from the mouse genome not only enhanced growth and protection of the intestinal mucosa but also promoted epithelial repair after acute injury. We also demonstrate that loss of *Cdr1as* causes miRNA deregulation in the intestinal epithelium and that disrupted expression of miR-195 in *Cdr1as^–/–^* mice primarily results from its destabilization and defects in biogenesis. These findings expand our knowledge of the pathobiological function of circRNAs in the intestinal epithelium and represent a major conceptual advance linking *Cdr1as* with intestinal mucosal defense and regeneration. Since the levels of *Cdr1as* increased in human intestinal mucosal tissues with injury/erosions and inflammation, our results suggest that a deregulated *Cdr1as*/miR-195 axis may play an important role in the pathogenesis of chronic mucosal injury, delayed repair, and impaired renewal of the intestinal epithelium in patients with various critical disorders.

The results reported here provide the first evidence to our knowledge showing the biological role of *Cdr1as* in maintaining the intestinal epithelium homeostasis. Mouse genetic models created using CRISPR/Cas9 showed that the *Cdr1as*-deficient epithelium of the small intestine displayed enhanced mucosal renewal that was associated with an increase in the numbers of Paneth, goblet, and tuft cells. Deletion of the *Cdr1as* locus failed to change the levels of autophagy-associated proteins in the intestinal mucosa but it enhanced the epithelial barrier function, as indicated by a decrease in gut permeability to FITC-dextran in *Cdr1as^–/–^* mice. Consistent with our findings, loss of *Cdr1as* in brain tissues upregulates immediate-early response genes such as *Fos*, *Arc*, and *Egrs*, and affects brain functions such as excitatory synaptic transmission and neuropsychiatric-like alteration in the behavior of *Cdr1as^–/–^* mice (21). *Cdr1as* also modulates the inflammatory phenotype in macrophages (37) and enhances differentiation of goat skeletal muscle satellite cells (38). However, opposing evidence also exists supporting proproliferative and oncogenic properties for *Cdr1as*, primarily in studies conducted in cultured cancer cells. It was reported that increasing the levels of cellular *Cdr1as* promoted proliferation of cholangiocarcinoma and adenocarcinoma cells (39, 40) and induced metastasis of lung squamous carcinoma cells (41). Thus, it appears that *Cdr1as* can promote or repress proliferation, likely depending on the cell type, the presence of other factors such as its target miRNAs, and the growth conditions.

Our results also indicate that decreasing the levels of cellular *Cdr1as* by removing the *Cdr1as* locus enhances injury-induced regeneration of the intestinal epithelium, an effect that is mediated, at least in part, via interaction with miR-195. We observed a remarkable enhancement of epithelial repair after acute injury in *Cdr1as^–/–^* mice, as evidenced by an induced recovery of damaged mucosa in the small intestine after exposure to mesenteric I/R. *Cdr1as^–/–^* mice also exhibited an increased tolerance to DSS-induced injury/erosions and inflammation in the colon. When comparing the levels of miRNAs between littermates and *Cdr1as^–/–^* mice, miR-195, a well-defined biological repressor of gut epithelial renewal (34–36), was consistently and markedly downregulated in the *Cdr1as*-deficient epithelium. This repression of miR-195 expression in *Cdr1as^–/–^* mice is crucial for controlling epithelial regeneration, since *Cdr1as*-mediated inhibition of epithelial repair after wounding in vitro and disrupted growth of intestinal organoids ex vivo were almost totally abolished by miR-195 silencing. In support of the present findings, several circRNAs were recently found to play an important role in maintaining the intestinal epithelial integrity and defense (42–44). *circHIPK3* enhanced intestinal epithelial repair after acute injury and promoted constant renewal of the epithelium by reducing miR-29b function, but its tissue levels in intestinal mucosa decreased significantly in patients with IBD (14). *circMaml2* facilitated healing of the intestinal mucosa via recruiting PTBP1 and regulating Sec62 (42)*,* and *circPABPN1* modulated intestinal epithelium host defense by altering translation of autophagy-associated protein ATG16L1 through interaction with HuR (33).

Another interesting finding from this study is that *Cdr1as* is involved in the regulation of miRNA biogenesis and upregulates miR-195 expression partially by increasing the stability of pre-miR-195. Loss of the *Cdr1as* locus in mice was found to decrease the levels of Dicer and TRBP proteins that operate in complexes and control miRNA processing in the cytoplasm (45, 46). To the best of our knowledge, this is the first evidence that *Cdr1as* is required for Dicer and TRBP expression in the intestinal epithelium. Since altered levels and activity of Dicer and its binding partner TRBP affect miRNA processing from the precursors to mature miRNAs (47), our results suggest that decreased levels of tissue Dicer and TRBP observed in the intestinal mucosa of *Cdr1as^–/–^* mice may deregulate miR-195 maturation in the cytoplasm. On the other hand, loss of *Cdr1as* decreased the levels of pre-miR-195 in the intestinal epithelium, although it failed to alter abundances of ADAR1, DDX17, and Drosha proteins that form the Drosha complex and process miRNAs from primary transcripts to precursors in the nucleus (48). Interestingly, ectopically expressed *Cdr1as* stabilized pre-miR-195 in cultured IECs, suggesting that decreased levels of pre-miR-195 in the *Cdr1as*-deficient epithelium partially results from its destabilization. Compared with miRNA biogenesis, turnover of miRNAs has received only limited attention to date, but several studies have revealed that accelerated or regulated miRNA turnover is crucial for controlling cellular miRNA accumulation and function (47–49). miR-29b decays more quickly in cycling cells than in cells arrested in mitosis (50), and cytomegalovirus infection decreases miR-27a levels by inducing its degradation (51). We have reported that lncRNA *uc.173* promotes renewal of the intestinal epithelium by inducing degradation of pri-miR-195 (31).

Identifying the function of the *Cdr1as*/miR-195 axis in intestinal epithelium is of particular importance from a clinical point of view because human intestinal mucosa with injury/erosions and inflammation from patients with IBD and sepsis exhibited increased levels of *Cdr1as* that were associated with the deregulated expression of many miRNAs, including miR-195, as reported previously (31, 52). miR-195 is an evolutionarily conserved miRNA with a variety of distinct cellular functions, and its expression levels increase markedly in chronic and unhealed injury and intestinal mucosa with growth inhibition (31, 35). miR-195 exerts its regulatory functions by suppressing the expression of different proteins (CDK4, CDK6, WEE1, IGF2 receptor, STIM1, and DCLK1) essential for repair and constant renewal of the intestinal epithelium (35, 53, 54). In this study, we uncovered a pivotal role for *Cdr1as* in coordinating intestinal epithelium homeostasis by altering miR-195 function. *Cdr1as^–/–^* mice displayed increased growth and enhanced repair after acute injury in the intestinal mucosa and this stimulatory effect is mediated primarily through interaction with miR-195. We also found that *Cdr1as* upregulated miR-195 expression at multiple levels, including cytoplasmic processing and pre-miR-195 stability.

In summary, our results indicate that interactions between *Cdr1as* and miR-195 are important for maintaining homeostasis of the intestinal epithelium and suggest that *Cdr1as* and miR-195 represent possible therapeutic targets to protect intestinal epithelial integrity and enhance mucosal repair after injury in patients with critical illness.

## Methods

### Chemicals and cell culture.

Caco-2 cells were purchased from the American Type Culture Collection and were maintained under standard culture conditions (22). The culture medium and FBS were purchased from Invitrogen and the chemicals from Sigma-Aldrich. Antibodies recognizing PCNA (catalog 2586), Cdk6 (catalog 3136), mucin 2 (catalog 88686), p62 (catalog 5114), ATG5 (catalog 12994), ATG12 (catalog 4180), ATG16L1 (catalog 8089), beclin 1 (catalog 3738), LC3 (catalog 12741), NOD1 (catalog 3545), GAPDH (catalog 2118s) (all from Cell Signaling Technology); E-cadherin (catalog 610182, BD Biosciences), occludin (catalog 33-1500), HuR (catalog sc-5261, Santa Cruz Biotechnology), beclin 2 (catalog PA1-46312, Invitrogen), NOD2 (catalog 14-5858-82, Invitrogen), Hsc70 (catalog ADI-SPA-815-F, Enzo Life Sciences), and GAPDH (catalog sc-32233, Santa Cruz Biotechnology). The secondary antibody conjugated to horseradish peroxidase was from Sigma-Aldrich. All antibodies utilized in this study were validated for species specificity. Antibody dilutions used for Western blots of PCNA, Cdk6, E-cadherin, occludin, mucin 2, p62, HuR, ATG5, ATG12, ATG16L1, beclin 1, beclin 2, LC3, NOD1, NOD2, Hsc70, and GAPDH were 1:800 or 1:1000 (primary antibody) and 1:2000 (secondary antibody). Relative protein levels were analyzed by using the Bio-Rad Chemidoc and XRS system equipped with Image Lab software (version 4.1). We also utilized the “Quantity tool” to determine the band intensity volume; the values were normalized to the internal loading control, GAPDH.

### Intestinal organoid culture and plasmid construction.

Isolation and culture of primary enterocytes were conducted following the method described previously (28, 55). Briefly, primary crypts were released from the small intestinal mucosa in mice; isolated crypts were mixed with Matrigel (BD Biosciences) and cultured in IntestiCult organoid growth medium (STEMCELL Technologies). The levels of DNA synthesis were measured by BrdU incorporation, and the growth of organoids was examined by measuring the surface area of organoid horizontal cross sections using the NIS-Elements AR4.30.02 program.

The recombined Lenti-*Cdr1as* vector was custom made by AMSBIO, in which intron–*Cdr1as* locus–intron/GFP expression was under the control of the suCMV promoter (24). Lenti-*Cdr1as* and Lenti-Con vectors were packaged in lentiviral production cells, concentrated by ultracentrifugation, resuspended in PBS, and used to increase *Cdr1as* in vitro and ex vivo, as described previously (8, 24).

### Generation of Cdr1as^–/–^ mice and animal experiments.

To create a loss-of-function mouse model, the *Cdr1as* locus was deleted from the mouse genome using the CRISPR/Cas9 approach (21). Briefly, 1-cell embryos were microinjected with Cas9 protein and 2 single-guide RNAs (sgRNAs) designed to target sequences upstream of *Cdr1as* splice sites, and the *Cdr1as^–/–^* strain was generated on the pure C57BL/6J background. Different sgRNAs were selected and synthesized by Synthego Inc. (Figure 2A and Supplemental Figure 1), and the sgRNA CRISPR/Cas9 efficiency was validated with the expertise provided by Genome Modification Facility at Harvard University using an ex vivo validation approach. Cas9 protein obtained from IDT and the synthetic sgRNAs were introduced into mouse embryos, and then the embryos were cultured to blastocysts. Each embryo was individually lysed, denatured, and PCR was performed followed by T7E1 assay to confirm genome editing at the *Cdr1as* locus. For microinjections, zygotes were obtained by mating of C57BL/6J males with superovulated C57BL/6J females (Jackson Laboratory). Genotyping analysis showed that there were 21 *Cdr1as^–/–^* mice selected from a total of 44 mice generated by CRISPR/Cas9 (Supplemental Figure 1D) and they were housed in a pathogen-free animal facility at the Baltimore VA Medical Center.

Animals were deprived of food but allowed free access to tap water for 24 hours before experiments. To generate the model of CLP-induced injury, age-matched male and female mice were anesthetized by Nembutal, and CLP was performed as described previously (23). Forty-eight hours after CLP, two 4-cm segments taken from the middle of the small intestine were removed from each animal as described previously (14, 22). To generate the model of small intestinal I/R-induced injury, mice were anesthetized, and a midline abdominal incision was performed. Mice were exposed to 30-minute superior mesenteric artery ischemia, followed by reperfusion for 2 hours as described previously (56). Sham operation for controls only involved laparotomy without mesenteric ischemia. To generate the colonic mucosal injury model, mice were fed with 3% DSS dissolved in drinking water for 7 consecutive days, as previously reported (22, 24).

### Studies using human tissues.

Human tissue samples were obtained from surplus discarded tissues from the Department of Surgery, University of Maryland Health Science Center (Baltimore, Maryland) and commercial tissue banks (BioIVT). Total RNA was isolated from the colonic mucosa in patients with UC and from the ileal mucosa in patients with CD by using the RNeasy mini kit (Qiagen), as described previously (14). For RNA-FISH assay, dissected and opened intestines were mounted onto a solid surface and fixed in formalin and paraffin, as carried out according to the method described in Xiao et al. (27).

### Histology, immunofluorescent staining, and RNA-FISH assay.

Dissected and opened intestines were mounted onto a solid surface and fixed in formalin and paraffin. Sections of 5 μm thickness were stained with H&E for general histology. Using a micrometer eyepiece, the overall length of villus and crypts and microscopic mucosal injury/erosions of each section were measured as described previously (14, 22). The immunofluorescent staining procedure was carried out according to the method we have described previously (8, 28). For experiments using mucosal tissue samples from mice, more than 5 slides (5 μm thickness section) in each tissue sample were prepared for immunofluorescent staining. For studies in cultured intestinal organoids, the slides were fixed in 3.7% formaldehyde in PBS and rehydrated. All slides were incubated with a primary antibody recognizing Ki67, lysozyme, DCLK1, or E-cadherin in blocking buffer overnight and then incubated with secondary antibody conjugated with Alexa Fluor 594 (Molecular Probes) for 2 hours at room temperature. After rinsing 3 times, the slides were incubated with DAPI (Molecular Probes) at a concentration of 1 μM for 10 minutes to stain cell nuclei. Finally, the slides were washed, mounted, and viewed through a Zeiss confocal microscope (model LSM700). Slides were examined in a blinded fashion by coding them, and only after examination was complete were they decoded. Images were processed using Photoshop software (Adobe).

The RNA-FISH assay was performed with the ISH optimization kit from Exiqon, as described previously (27, 31). Briefly, the slides were deparaffinized and then incubated with proteinase-K. After washes with PBS, the slides were dehydrated and hybridized with 25 nM fluorescent LNA probe for 1 hour at 60°C. The slides were washed with SSC buffers and PBS, covered with cover slides, and then processed using a Zeiss confocal microscope.

### Q-PCR analysis and miRNA microarray.

Total RNA was used in RT and PCR amplification reactions as described previously (27, 57). Q-PCR analysis was performed using the Step-one-plus System with specific primers, probes, and software (Applied Biosystems). To measure circRNA levels, total RNA was digested with RNase R to remove all linear RNAs; after RT, the cDNA library was used for amplification (Q-PCR analysis) using primer pairs that spanned the circularization junction. To examine the subcellular distribution of RNA, nuclear and cytoplasmic fractions from Caco-2 cells were isolated and purified with the SurePrep Nuclear/Cytoplasmic RNA Purification Kit from Fisher Bioreagents, following the manufacturer’s instructions. The levels of β-tubulin (a cytoplasmic protein marker) and lamin B (a nuclear protein marker) were examined in the 2 fractions to ascertain the purity of the cytoplasmic and nuclear preparations. Total RNA was isolated from nuclear and cytoplasmic fractions, and the levels of *Cdr1as* and lncRNAs were examined by Q-PCR analysis, as described previously (8, 31). For miRNA array studies, total RNA was purified with a miRCURY RNA isolation kit, and miRCURY LNA array of miRNA profiling services was performed by Arraystar. *Cdr1as* levels were quantified by Q-PCR analysis by using a SYBR Green PCR assay.

### Immunoblotting analysis.

Whole-cell lysates were prepared using 2% SDS, sonicated, and centrifuged at 4°C for 15 minutes. The supernatants were boiled and size-fractionated by SDS-PAGE. After the blots were incubated with primary and then secondary antibodies, immunocomplexes were detected by using chemiluminescence.

### Assays of gut permeability.

FITC-dextran dissolved in water (Sigma-Aldrich; 4 kDa, 600 mg/kg) was administered to mice via gavage as described previously (32). Blood was collected 4 hours thereafter via cardiac puncture. The serum concentration of the FITC-dextran was determined using a plate reader with an excitation wavelength at 490 nm and an emission wavelength of 530 nm. The concentration of FITC-dextran in sera was determined by comparison to a FITC-dextran standard curve.

### Measurement of epithelial repair in vitro.

The epithelial injury model and repair assays were performed as described previously (14, 35). Cells were plated at 6.25 × 10^4^/cm^2^ in DMEM containing FBS on 60-mm dishes thinly coated with Matrigel according to the manufacturer’s instructions (BD Biosciences) and were incubated as described for stock cultures. Cells were fed on day 2, and the monolayer was wounded by removing part of the monolayer on day 4; repair was assayed at different times after wounding by using NIS-Elements AR v4.30.02 image analysis. All experiments were carried out in triplicate, and the results are reported as the percentage of wound width covered.

### Statistics.

All values are expressed as the mean ± SEM. Unpaired, 2-tailed Student’s *t* test was used when indicated. When assessing multiple groups, 1- or 2-way ANOVA was utilized with Tukey’s post hoc test (57). The statistical software used was GraphPad Prism 9.0. For nonparametric analysis rank comparison, the Kruskal-Wallis test was conducted. A *P* value of less than 0.05 was considered significant.

### Study approvals.

All animal experiments were conducted in accordance with the NIH *Guide for the Care and Use of Laboratory Animals* (National Academies Press, 2011) and were approved by the Institutional Animal Care and Use Committee of the University Maryland School of Medicine and the Research and Development Committee at the Baltimore VA Hospital. The study using human tissues was approved by the University of Maryland Institutional Review Board.

### Data availability.

Primary data sets of miRNA expression profiles in the small intestinal mucosa of *Cdr1as^–/–^* and littermate mice as examined by microarray have been generated and deposited in NCBI (GEO GSE250489). All supporting data for each figure panel are available in the Supporting Data Values file. Any additional information required to reanalyze the data reported in this paper is available upon request.

## Author contributions

HKC and LX performed most experiments and summarized data. NH, JC, VY, CMC, BR, and JNR participated experiments in vivo, immunoprecipitation assays, and experiments conducted in intestinal organoids and cultured IECs. DJT and RK participated in experiments using human tissues and data analysis. MG participated in data analysis and edited the manuscript. JYW designed experiments, analyzed data, prepared figures, and drafted the manuscript. All authors reviewed the final manuscript.

## Supplementary Material

Supplemental data

Unedited blot and gel images

Supporting data values

## Version 1 01/16/2024

In-Press Preview

## Version 2 02/22/2024

Electronic publication
