# Supplementary material for: Circular RNA Cdr1as inhibits proliferation and delays injury-induced regeneration of the intestinal epithelium
Source: JCI Insight. 2024 Jan 16;9(4):e169716. doi: 10.1172/jci.insight.169716 (PMC11143936; doi:10.1172/jci.insight.169716)
Supplement: Supplemental data [file jciinsight-9-169716-s034.pdf]

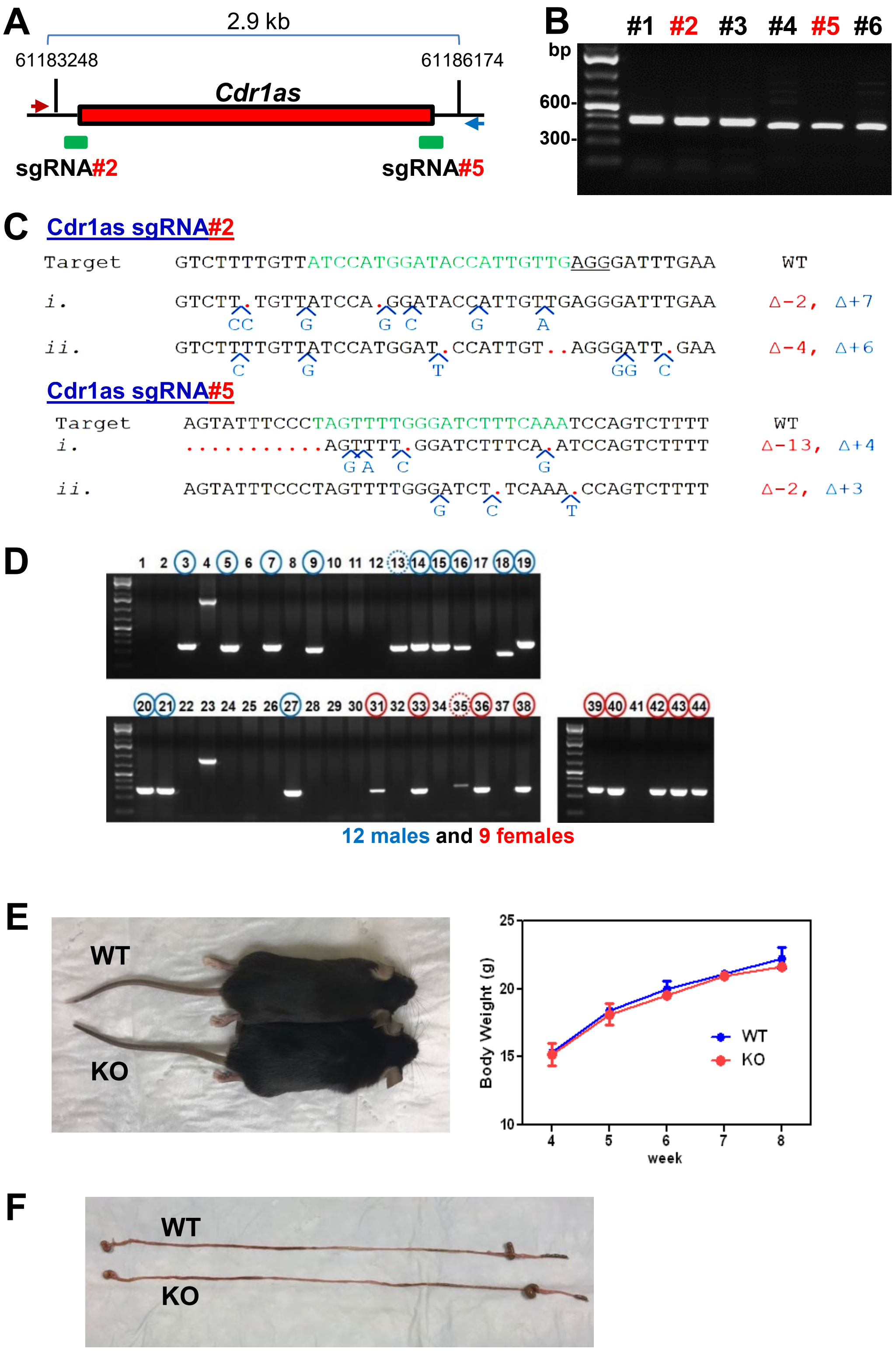

Supplementary Figure 1. Generation of Cdr1as knockout mice by CRISPR-Cas9. (**A**) The *Cdr1as* locus that was deleted. (**B**) PCR results from *ex vivo* validation in embryos microinjected with sgRNA and Cas9 enzyme. (**C**) DNA sequences of embryo microinjected with sgRNA #2 and #5. Green: sgRNA target; Δ-: deleted mutation; Δ+: insertional mutation. (**D**) Genotyping analysis showed that there were 21 Cdr1as knockout (KO) mice from total 44 mice generated by CRISPR-Cas9 technique. (**E,F**) Mouse, body weights, and gastrointestinal gross in littermate and Cdr1as$^{-/-}$ mice (n = 5).

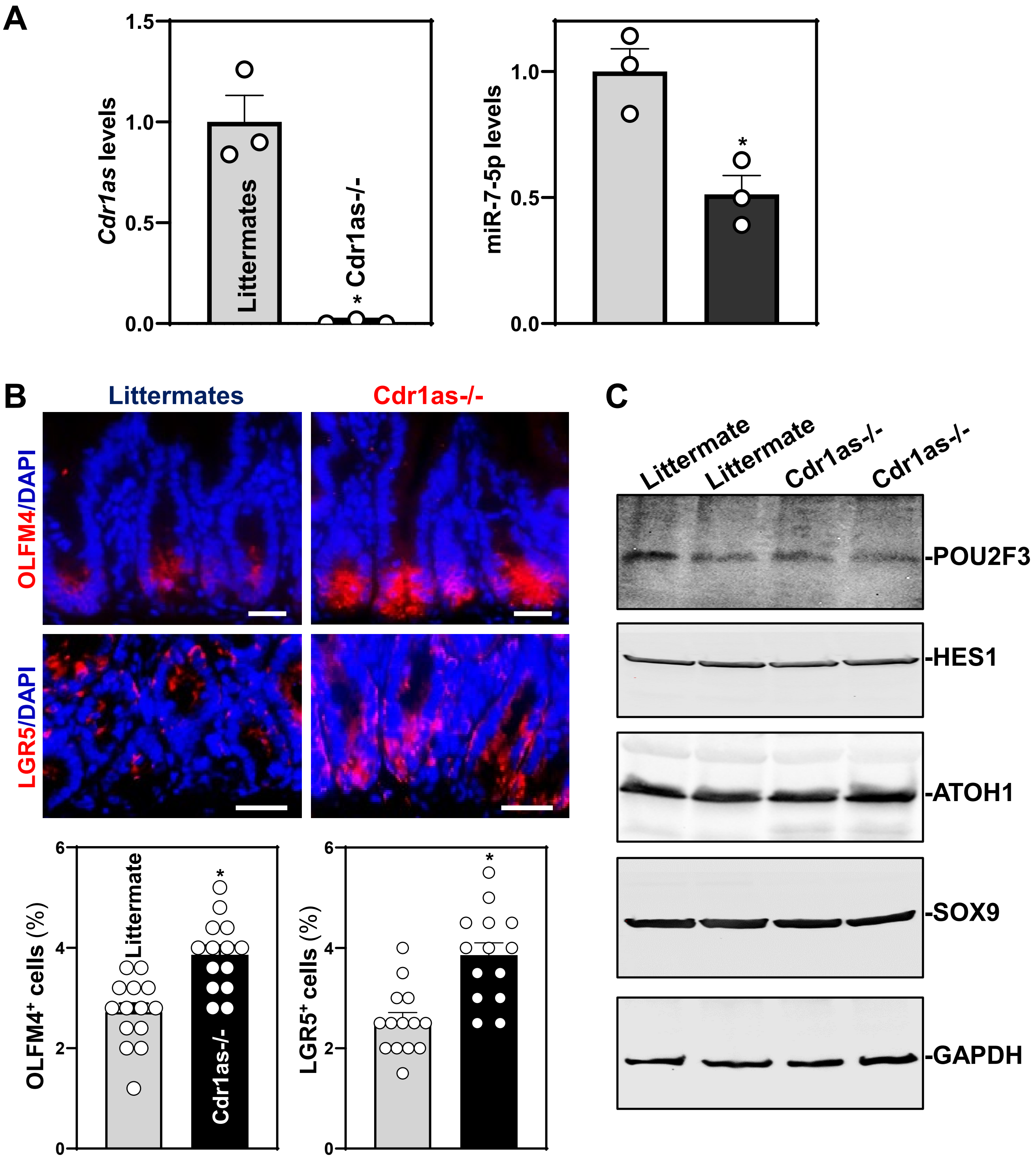

Supplementary Figure 2. (***A***) Levels of *Cdr1as* and miR-7-5p in the brain tissues of littermate and cdr1as-/- mice as measured by Q-PCR assay. Values are the means ± SEM (n = 3). * $P < 0.05$ compared with littermates. (***B***) Changes in ISC activity in the small intestinal mucosa after Cdr1as deletion, as examined by immunohistochemistry staining. ISCs were marked by OLFM4 (top) and LGR5 (bottom). scale bar: 25 µm. * $P < 0.05$ compared with control littermates (n = 10). (***C***) Immunoblots of POU2F3, HES1, ATOH1, and SOX9 in the small intestinal mucosa of littermate and Cdr1as-/- mice. Three separate experiments were performed and showed similar results.

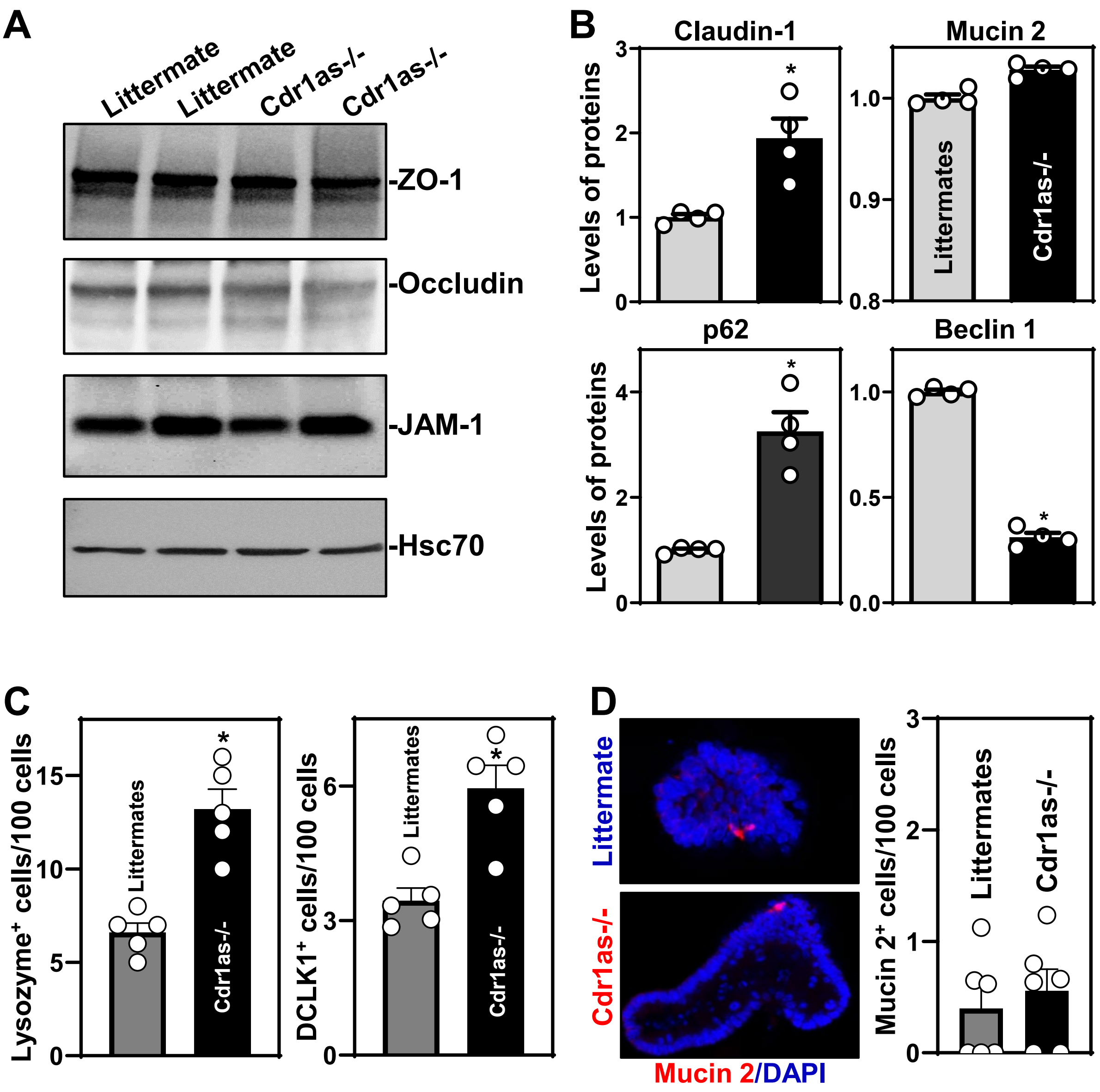

Supplementary Figure 3. (***A***) Immunoblots of ZO-1, occludin, and JAM1 in the small intestinal mucosa of littermates and Cdr1as$^{-/-}$ mice. Three separate experiments were performed and showed similar results. (***B***) Quantitative analysis derived from densitometric scans of immunoblots of claudin-1, mucin 2, p62, and beclin 1 in the small intestinal mucosa described in **Figure 3A**. Values are the means ± SEM (n = 4). **P* < 0.05 compared with control littermates. (***C***) Quantitative data of lysozyme-positive cells (*left*) and DCLK1-positive cell (*right*) in intestinal organoids described in **Figure 4E,F**. * *P* < 0.05 compared with the organoids generated from littermate mice (n = 5). (***D***) Goblet cells (marked by mucin 2) in intestinal organoids as described in **Figure 4E**. Values are the means ± SEM (n = 6)

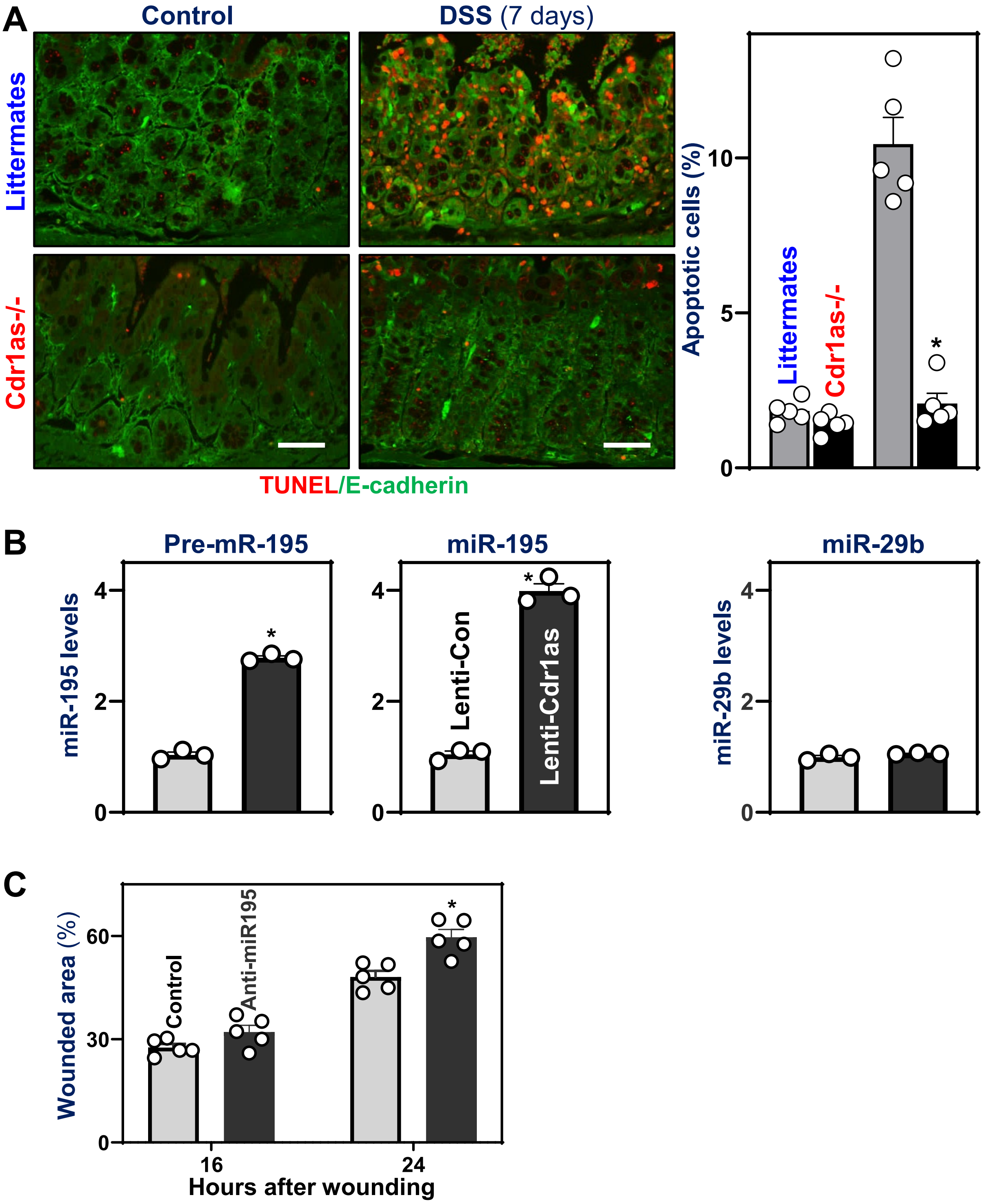

Supplementary Figure 4. (***A***) Apoptotic cells in the colonic mucosa of littermate and Cdr1as-/- mice treated with 3% DSS in drinking water for 7 days (DSS), as measured with TUNEL staining. scale bar: 25 µm. Values are means + SEM (n = 5). * P < 0.05 compared with littermates. (***B***) Levels of pre-miR-195, miR-195, and miR-29b in Caco-2 cells 72 h after infection with the lentiviral *Cdr1as* expression vector (Lenti-*Cdr1as*) or control lentiviral vector (Lenti-Con) as measured by Q-PCR. Values are the means ± SEM (n = 3). * P < 0.05 compared with Lenti-Con. (***C***) Summarized data of epithelial repair after wounding in cells treated with lenti-Con alone or lenti-Con plus anti-miR195. * P < 0.05 compared with Len-Con alone (n = 5).
