# Supplementary figures and images for: Circular RNA Cdr1as inhibits proliferation and delays injury-induced regeneration of the intestinal epithelium
Source: JCI Insight. 2024 Jan 16;9(4):e169716. doi: 10.1172/jci.insight.169716 (PMC11143936; doi:10.1172/jci.insight.169716)

Figure 3A, Left

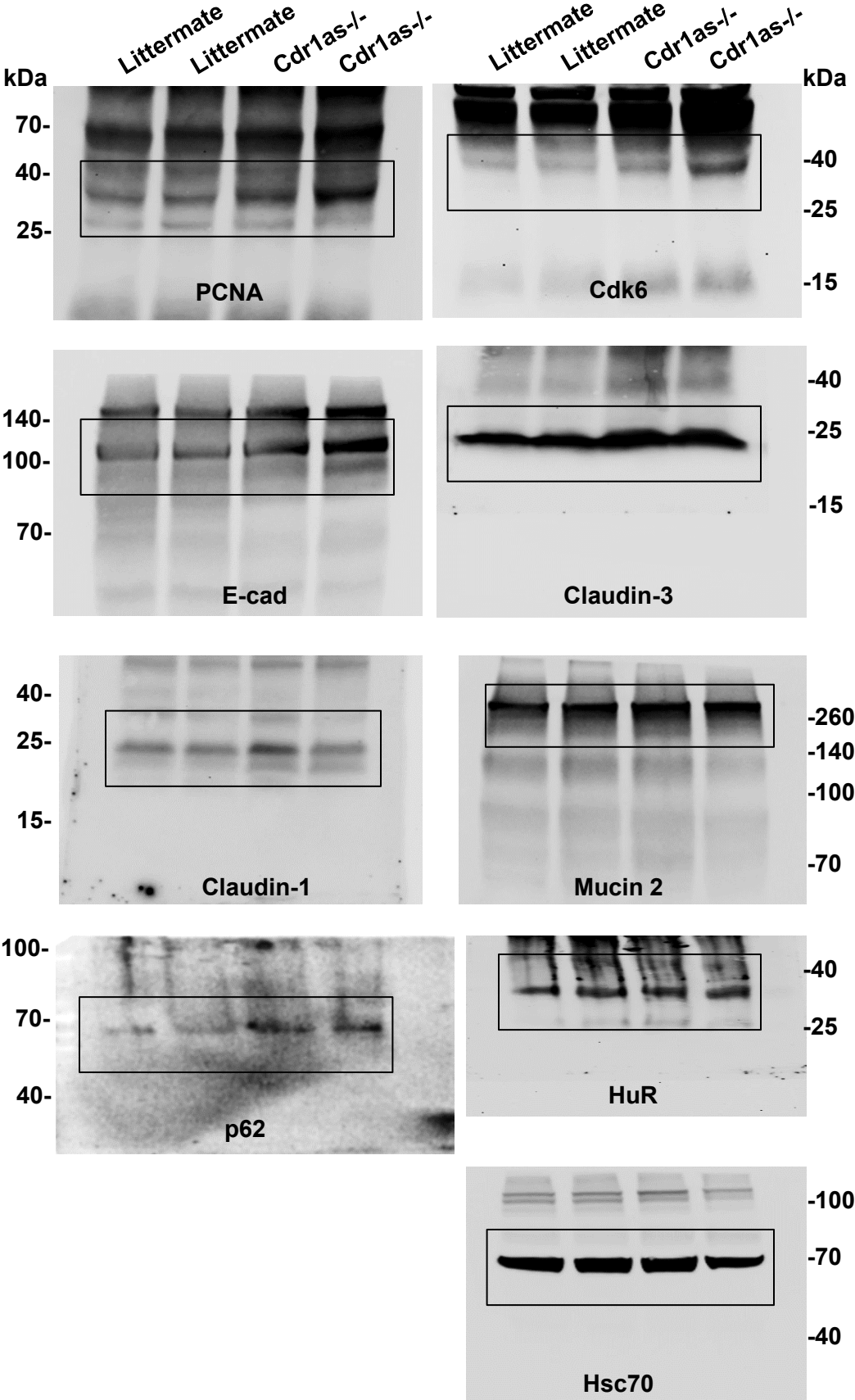

Figure 3A, Right

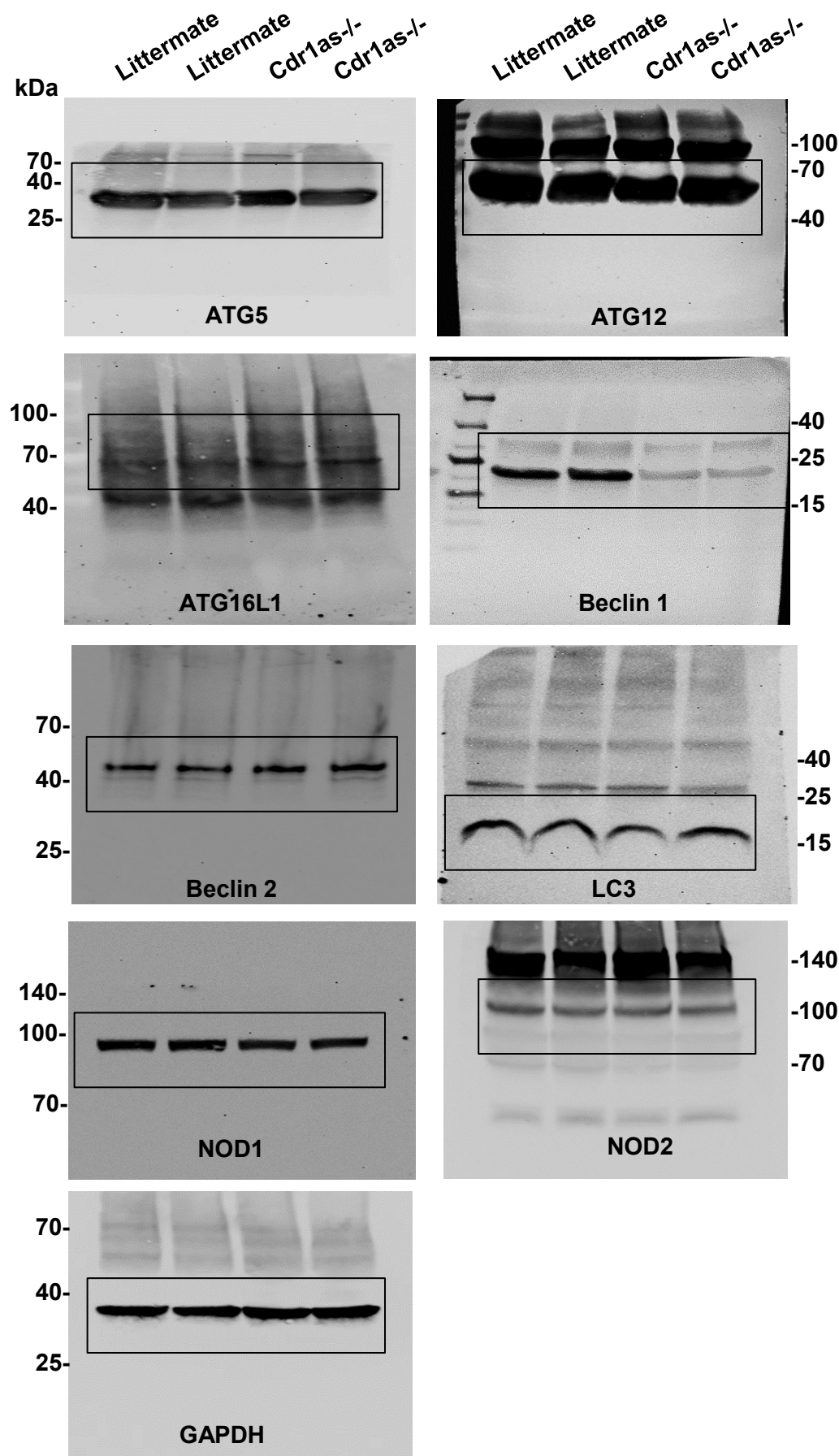

Figure 8C

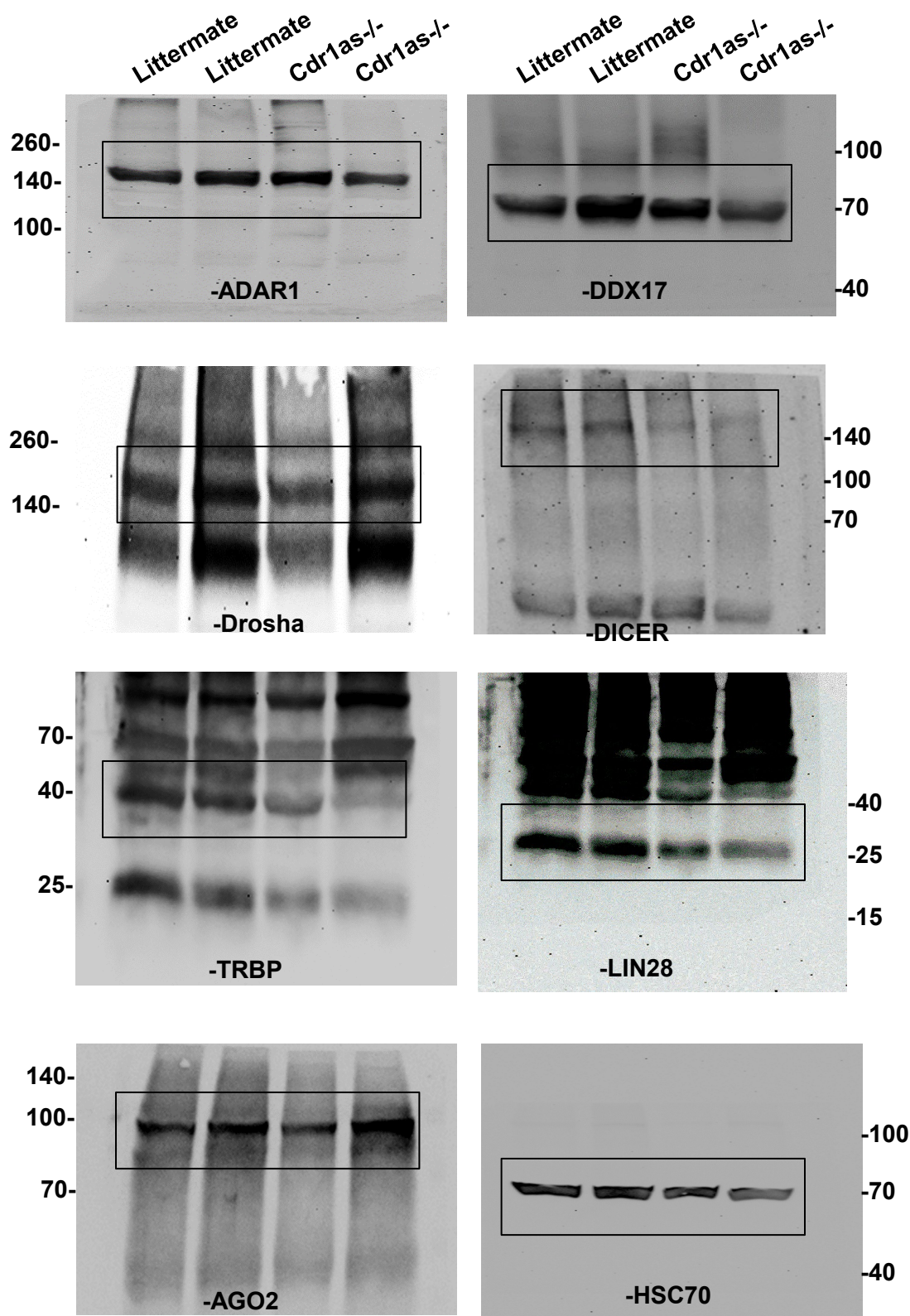

Supplementary Figure 2C

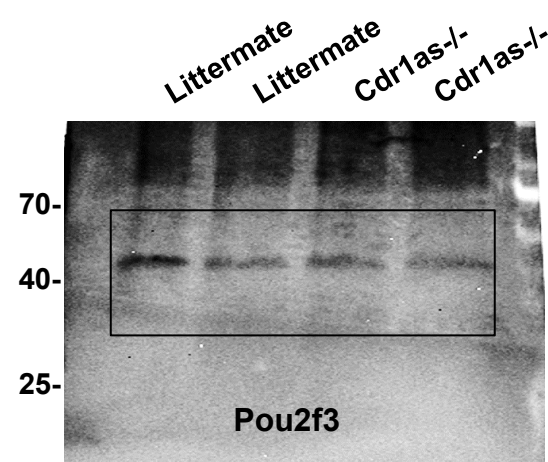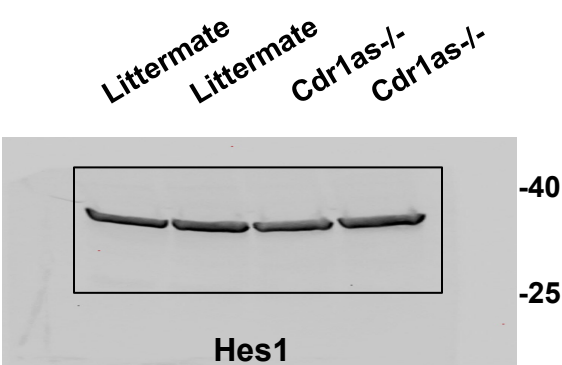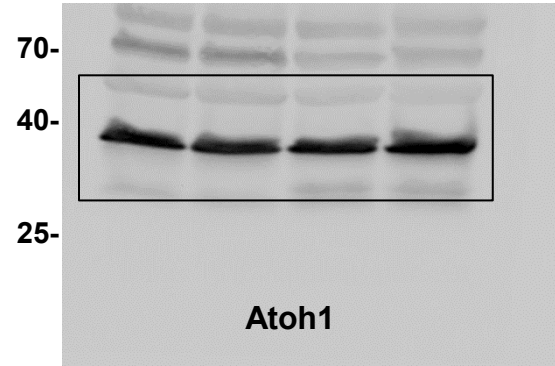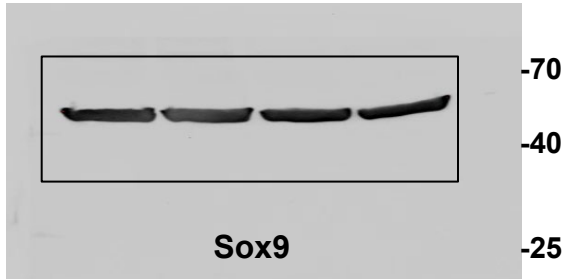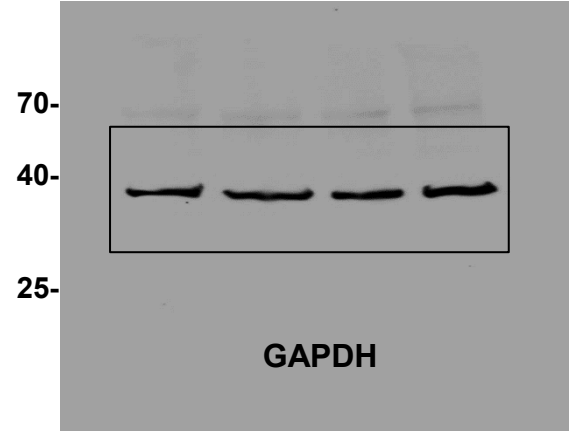

Supplementary Figure 3A

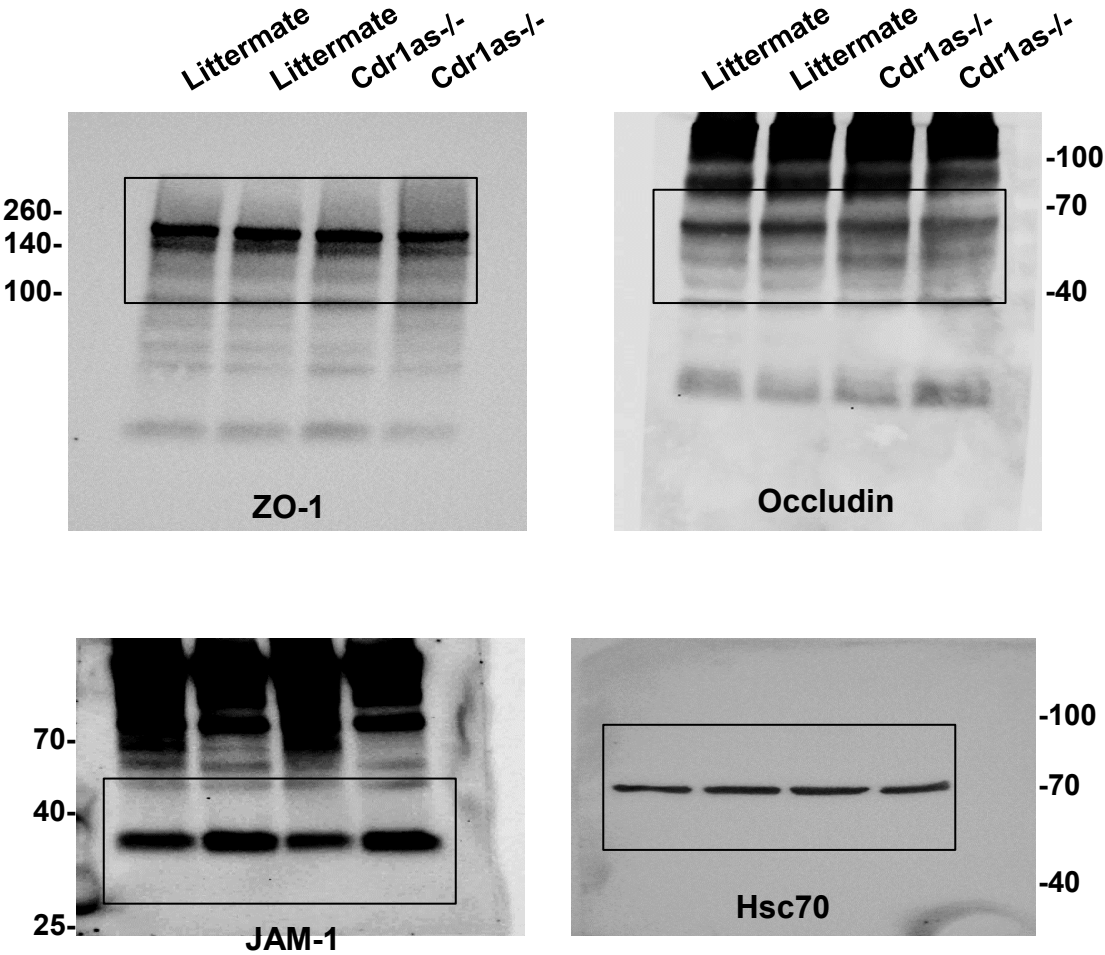

Supplement: Unedited blot and gel images [file jciinsight-9-169716-s035.pdf]
